# Low-dimensional population dynamics in the brainstem gate REM sleep

**DOI:** 10.1038/s41593-026-02314-z

**Published:** 2026-05-25

**Authors:** David E. Lozano, Jiso Hong, Xi Jin, Joseph A. Stucynski, Christian K. Machens, Shinjae Chung, Franz Weber

**Affiliations:** 1 Department of Neuroscience, Chronobiology and Sleep Institute, Perelman School of Medicine, University of Pennsylvania, Philadelphia, PA USA; 2 Champalimaud Neuroscience Programme, Champalimaud Foundation, Lisbon, Portugal

**Keywords:** Circadian rhythms and sleep, Neural circuits

## Abstract

Rapid-eye-movement (REM) sleep is generated in the brainstem, but the brainstem population dynamics that drive transitions to REM sleep remain largely unknown. Here, combining mouse Neuropixels recordings and dimensionality reduction, we found that population activity in the midbrain and pons is dominated by two components, one of which captures strong infraslow fluctuations in neural activity. During transitions from non-REM (NREM) to REM sleep, the population activity followed a stereotypic trajectory that was preceded by an increase in the infraslow component. Our analysis revealed—across all brainstem areas—subpopulations of REM sleep-activated and REM sleep-inhibited neurons with opposing infraslow dynamics and diverging ramping activity between REM sleep episodes, reinforced through antagonistic functional connections. Activation of REM sleep-promoting medullary neurons rapidly enhanced the infraslow component, whose strength gated the ability of upstream circuits to induce REM sleep. Collectively, our results identify a population-level mechanism for gating REM sleep, suggesting that NREM-to-REM sleep transitions are coordinated by low-dimensional, antagonistic brainstem dynamics.

## Abstract (web summary)

Lozano et al. show that REM sleep is gated by low-dimensional brainstem network dynamics, in which opposing neuron populations across the midbrain and pons determine when transitions into REM sleep can occur.

## Main

As the sleeping brain transitions from non-REM (NREM) to REM sleep, it undergoes dramatic changes, switching from a state of generally low activity to a metabolically demanding state with heightened activity in multiple brain areas spanning from the brainstem to the cortex^1^. Over the past decade, studies have identified distinct REM sleep-regulatory neuron populations within and beyond the brainstem and mapped their connectivity with unprecedented detail^1–3^. Nodes within this circuit map are typically categorized as either REM sleep-promoting (REM-on) or REM sleep-suppressing (REM-off) neurons. However, despite having this detailed circuit map, we lack an understanding of the activity dynamics on the population level that govern transitions into and out of REM sleep and consequently drive the ultradian alternation between NREM and REM sleep (NREM–REM cycle).

In mice, REM sleep recurs on a timescale of several minutes^4,5^. Two processes have been proposed to govern the timing of NREM→REM transitions. The propensity to enter REM sleep on the ultradian timescale is thought to be driven by the accumulation of REM sleep-specific homeostatic pressure in the absence of REM sleep^4,6–8^. In addition, recent work suggests that faster infraslow (~1 min) oscillations in the electroencephalogram (EEG) correlate with recurring windows of opportunity during which the chance for NREM→REM transitions is increased on a minute-to-minute basis^9,10^. Infraslow and ultradian modulations in neural activity have been described for several neural populations involved in REM sleep regulation: Noradrenergic neurons in the locus coeruleus, dorsal raphe (DR) serotonergic neurons and REM-on neurons in the dorsomedial medulla (dmM) show pronounced infraslow activity fluctuations^10–14^, whereas ultradian activity modulations in synchrony with the NREM–REM cycle have been observed in REM-off neurons in the DR^12^ and ventrolateral periaqueductal gray (vlPAG)^15^. However, it remains unknown whether these infraslow and ultradian dynamics are unique to specific subpopulations or are shared across broader populations of REM sleep-regulatory neurons. Consequently, we lack an understanding of how these two processes are intertwined at the population level in REM-regulatory areas and thereby affect the moment-to-moment propensity to enter REM sleep.

Previous work has identified multiple populations of REM-on neurons distributed across different areas within the brainstem, hypothalamus, amygdala and cortex^16–21^. Many REM-on neurons are inhibitory and, as a common circuit motif, project to midbrain areas containing REM-off neurons such as the vlPAG, DR and neighboring midbrain reticular nucleus (MRN, also referred to as DpMe)^10,16,22–25^. Electrophysiological studies in these areas identified large numbers of REM-off neurons with minimal activity during REM sleep^15,26–31^. Optogenetic or chemogenetic activation of REM-off populations, such as vlPAG/DpMe GABAergic neurons or DR serotonergic neurons, effectively suppressed NREM→REM transitions^15,26,32,33^, whereas inhibition or ablation of neurons in the vlPAG/DpMe promoted REM sleep^15,24,27,34–36^. Midbrain REM-off neurons are, therefore, thought to play a key role in gating REM sleep. Specifically, their inhibition by presynaptic REM-on neurons has been proposed to disinhibit the core circuits in pons and medulla that control the defining features of REM sleep (muscle atonia and rapid eye movements)^1,3^. However, it remains unknown how the activation of upstream REM-on neurons affects in vivo the population dynamics within midbrain and pontine areas to give rise to NREM→REM transitions.

Performing Neuropixels recordings in sleeping mice, we found that the population activity in midbrain and pontine areas involved in REM sleep regulation is low-dimensional and dominated by two components. The first component is strongly activated during REM sleep, whereas the second captures infraslow fluctuations in neural activity. NREM→REM transitions were preceded by a pronounced increase in this infraslow component. Across all regions, we identified neuron subpopulations with opposing dynamics along this dimension, spanning synaptic, infraslow and ultradian timescales. Optogenetic activation of REM-on neurons in the dmM rapidly increased the infraslow component, whose current strength influenced the capability of upstream circuits to trigger REM sleep. In contrast, excitation of wake-promoting neurons in the medulla strongly suppressed this component. In sum, these findings identify a key population component along which subpopulations with antagonistic infraslow and ultradian dynamics interact to gate NREM→REM transitions.

## Results

### Low-dimensional population activity in midbrain and pons during sleep

**Fig. 1:**
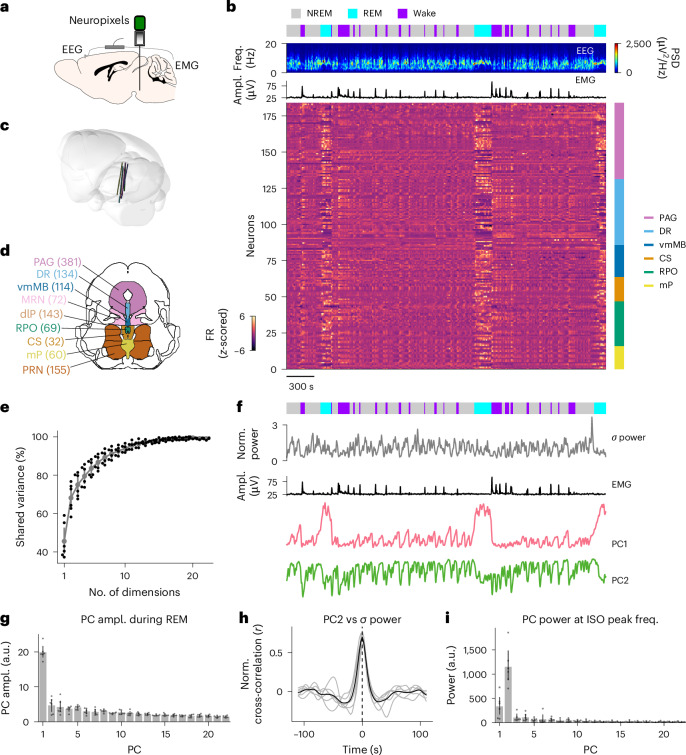
Low-dimensional population activity in midbrain and pons during sleep. **a**, Schematic illustrating Neuropixels recording in pons and midbrain areas of a C57BL/6J mouse. **b**, Example recording session, including hypnogram, normalized EEG spectrogram, EMG amplitude and heat map representing the firing rates of 185 simultaneously recorded neurons. Each row color-codes the *z*-scored firing rate of a single unit. Neurons are sorted by brain region and depth. The color bar on the right indicates the brain regions. FR, firing rate; PSD, power spectral density. **c**, Anatomical reconstruction of electrode tracts within the 3D Allen Mouse Brain Reference Atlas from six animals. **d**, Coronal brain schematic depicting recorded brain areas and number of neurons per area. **e**, Percentage of the shared variance in the population activity captured by increasing numbers of PCs. Gray dots indicate the mean across mice; error bars denote 95% confidence intervals (CIs); black dots represent individual mice (*n* = 6). **f**, Hypnogram, normalized EEG *σ* power (Methods), EMG amplitude and PC1/PC2 (both PCs are plotted using the same scaling). **g**, Mean absolute amplitude of PC1 to PC22 during REM sleep. Repeated-measures (RM) analysis of variance (ANOVA), *P* = 2.79 × 10^−60^, *F*(21,105) = 102.90; two-sided paired *t*-tests comparing PC1 with PC2, …, PC22, *P* < 0.0043; *n* = 6 mice. Dots indicate individual mice; error bars denote 95% CIs. See Supplementary Table 1 for statistical details. **h**, Normalized cross-correlation between PC2 and EEG *σ* power during NREM sleep (Methods). Black line indicates the mean across mice; gray lines denote individual mice. Two-sided one-sample *t*-test, *P* = 7.42 × 10^−7^, *T*(5) = 30.24; *n* = 6 mice. **i**, Power at ISO peak frequency for PC1 to PC22. For each animal, we calculated the peak ISO frequency in the EEG *σ* power (*f*_iso_, Extended Data Fig. 1e) and then determined for each PC its power at *f*_iso_. RM ANOVA, *P* = 2.19 × 10^−37^, *F*(21,105) = 33.90; two-sided paired *t*-tests with Bonferroni correction comparing PC2 with PC1, PC3, …, PC22, *P* < 0.041; *n* = 6 mice. Error bars indicate 95% CIs. See Supplementary Table 1 for statistical details. a.u., arbitrary units. Source data

**Extended Data Fig. 1:**
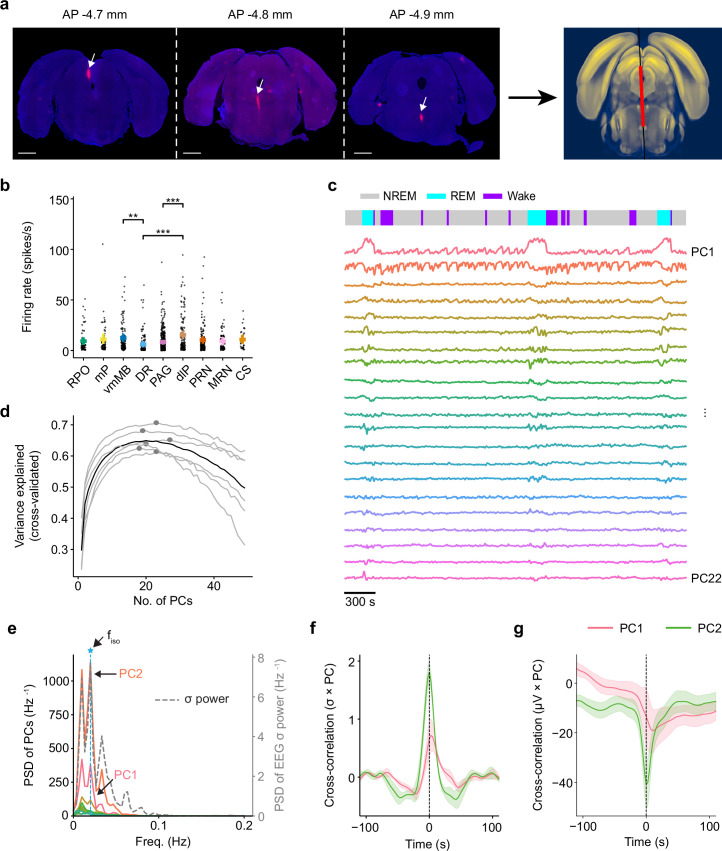
Electrode tract reconstruction and principal components (PCs). (**a**) Left, three histology pictures, arranged from anterior to posterior, showing parts of the DiI-labeled electrode tract (arrow heads) in a C57BL/6J wild-type (WT) mouse. Blue, 4’,6-diamidino-2-phenylindole (DAPI); red, DiI. Scale bars, 1 mm. Right, reconstructed electrode tract. The red line indicates the reconstructed tract within the 3D Allen Mouse Brain Reference Atlas along which units were recorded (**Methods**). The shown coronal section was aligned with the insertion angle of the electrode. (**b**) Average firing rates across brain regions. ANOVA, P = 8.21×10^−8^, F(8, 1151) = 6.17; two-sided t-tests with Bonferroni correction, DR vs dlP: P = 2.13e-05, T(222.68) = -5.14; DR vs vmMB: P = 0.00486, T(202.48) = -3.89; PAG vs dlP: P = 0.00087, T(174.76) = -4.34; RPO, n = 69; mP, n = 60; vmMB, n = 114; DR, n = 134; PAG, n = 381; dlP, n = 143; PRN, n = 155; MRN, n = 72; CS, n = 32 units from 6 mice. **P < 0.01, ***P < 0.001. Horizontal lines, mean; error bars, 95% CIs. See Supplementary Table 1 for full statistical details. (**c**) PC1 to PC22 for the same mouse as in Fig. 1b,f. For comparability, all PCs are plotted using the same scaling. (**d**) Relationship between cross-validated explained variance (CVEV) and number of PCs. The peak in the relationship between the CVEV and the number of PCs constitutes a lower bound on the dimensionality of the shared population variability (**Methods**). Gray dots indicate for each animal the peak CVEV. Black line, mean across mice; gray lines, individual mice. (**e**) Example illustrating how the strength of the infraslow modulation was computed for each PC in Fig. 1i. First, we determined the frequency f_iso_ (blue star) at which the power spectral density (PSD) of EEG σ power during NREM sleep peaked. Next, we calculated the PSD for each PC and determined its power at f_iso_. The PSDs of the PCs are colored using the same color-code as in **c**. (**f**) Cross-correlation of normalized EEG σ power with PC1 or PC2. The correlation was stronger for PC2 than PC1. Two-sided paired t-test comparing peak correlation between PC1 and PC2: P = 0.0072, T(5) = -4.37. Two-sided one-sample t-test whether peak correlation value differs from 0 (Bonferroni-corrected); PC1: P = 0.132, T(5) = 2.35; PC2: P = 3.31×10^−4^, T(5) = 10.07; n = 6 mice; shadings, s.e.m. (**g**) Cross-correlation between PC1 or PC2 and EMG amplitude. Two-sided one-sample t-test testing whether peak correlation values differ from 0 (Bonferroni-corrected); PC1: P = 0.136, T(5) = -2.32; PC2: P = 0.018, T(5) = -4.15; n = 6 mice; shadings, s.e.m. Source data

**Extended Data Fig. 2:**
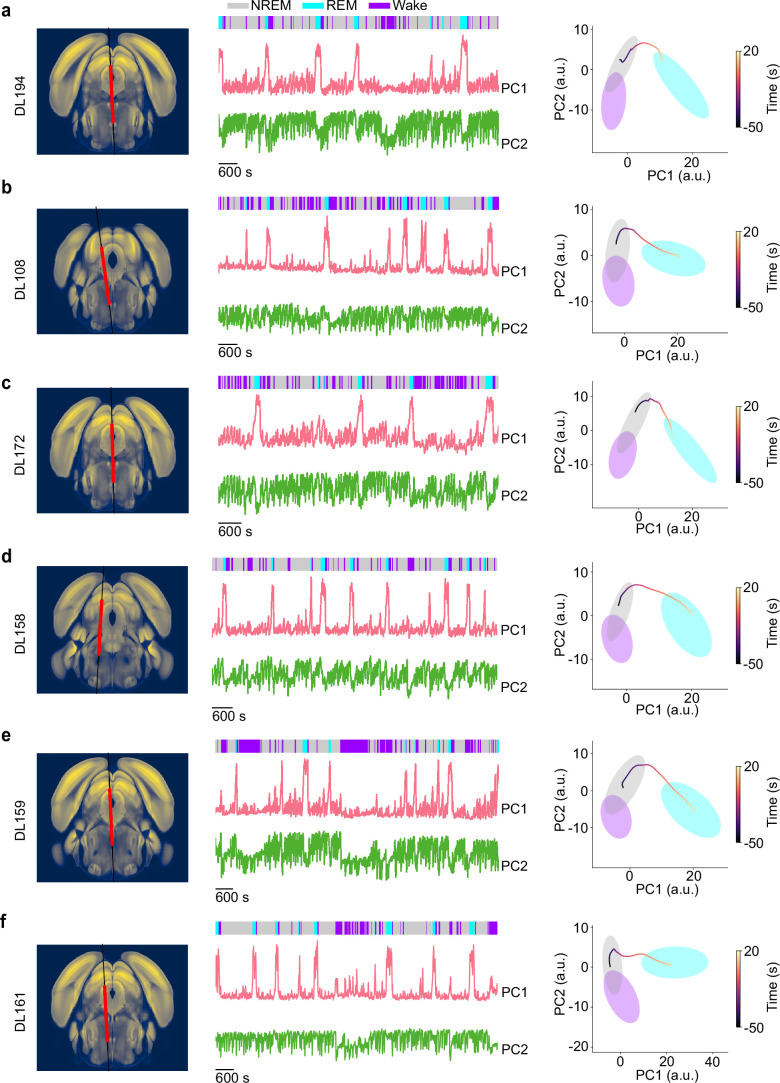
Electrode location, PCs and state space for each individual mouse. (**a-f**) Each row shows for one mouse in the WT data set the reconstructed electrode tract (left), PC1 and PC2 along with hypnogram (middle), and the resulting state space with the average NREM→REM trajectory (right). For comparability, PC1 and PC2 are plotted using the same scaling.

To investigate the population dynamics in REM sleep-regulatory brain areas in midbrain and pons, we performed high-density electrophysiological in vivo recordings using Neuropixels in head-fixed C57BL/6J mice, combined with simultaneous EEG and electromyogram (EMG) recordings for brain state classification (Fig. 1a,b and Methods). Mice were habituated to sleep well under head fixation (Methods and Supplementary Fig. 1). We verified that the amount of the different sleep–wake states is comparable in head-fixed and freely moving animals (Supplementary Fig. 2a). Previous work showed that, during NREM sleep, the EEG shows pronounced infraslow oscillations (ISOs), particularly within the *σ* (10–15 Hz) range^37^. The ISOs were indistinguishable between the two cohorts of mice (Supplementary Fig. 2b), despite a reduction in delta power during sleep (Supplementary Fig. 2c). At the end of each experiment, we reconstructed the location of the electrode tract and determined the brain region of each recorded neuron within the three-dimensional (3D) Allen Mouse Brain Reference Atlas^38,39^ (Fig. 1c, Extended Data Figs. 1a and 2 and Methods). Neuropixels recordings were performed during the light phase and lasted on average 2.56 h ± 0.10 h (mean ± s.e.m; *n* = 6 mice). In each animal, we simultaneously recorded the activity of 162 to 226 neurons, distributed across multiple brain areas including the periaqueductal gray (PAG), MRN and neighboring midbrain areas (summarized as the ventromedial midbrain or vmMB), multiple raphe nuclei in midbrain and pons (DR, nucleus raphe pontis (RPO) and superior central nucleus raphe (CS)) and pontine areas (pontine reticular nucleus (PRN), dorsolateral pons (dlP) and medial pons (mP); Fig. 1d, Supplementary Table 2 and Methods). The mean firing rates of the recorded neurons significantly varied across these areas (Extended Data Fig. 1b).

Visual inspection indicated that the firing rates of the individual neurons shared common activity patterns across the recorded areas, most prominently a strong modulation of their activity by the brain state and fluctuations on the minute (infraslow) timescale (Fig. 1b). To explicitly extract activity components shared across the population of simultaneously recorded neurons, we performed dimensionality reduction using principal component analysis (PCA). The total variance in the activity of a neural population comprises variability that is shared by multiple neurons (shared variance) and fluctuations that are unique to individual neurons (private variance). To estimate the fraction of the shared variance in the population activity explained by the principal components (PCs), we performed a cross-validated variant of PCA (Methods)^40–42^. The peak in the relationship between the number of PCs used to explain the neural activity and the cross-validated explained variance (CVEV) provides a lower bound on the maximal shared variance^40–43^. The CVEV peaked at 22.17 ± 1.62 PCs (*n* = 6 mice), explaining 65.54% ± 1.4% of the total variance (Extended Data Fig. 1d). The sharp rise of the CVEV curve with the number of PCs indicated a low dimensionality of the population activity during sleep. Accordingly, the first two PCs alone accounted for 67.85% ± 2.90% of the shared variance (Fig. 1e). The first principal component (PC1) was strongly activated during REM sleep (Fig. 1f) and compared with the other PCs, PC1 showed the largest amplitude during REM sleep (Fig. 1g and Extended Data Fig. 1c; see Supplementary Table 1 for statistical details). The second principal component (PC2) captured slow fluctuations in neural activity on the infraslow timescale (Fig. 1f). Cross-correlation analysis demonstrated that the EEG *σ* power and PC2 were indeed closely correlated during NREM sleep (Fig. 1h), and this correlation was stronger for PC2 than for PC1 (Extended Data Fig. 1f). Next, we calculated for each PC the power spectral density and determined its power at the peak frequency of the *σ* power ISOs (Extended Data Fig. 1e and Methods). We found that PC2 showed the strongest infraslow modulation (Fig. 1i and Extended Data Fig. 1c,e). Cross-correlating the PCs with the EMG amplitude further revealed a negative correlation between PC2 and the EMG activity (Fig. 1f and Extended Data Fig. 1g), indicating low activity of this component during wakefulness. Given the similarity of the first two PCs across mice (Extended Data Fig. 2) and that they accounted for two-thirds of the shared variance (Fig. 1e), we focused on these two PCs for the following analyses.

### Stereotypic NREM→REM transitions in state space

**Fig. 2:**
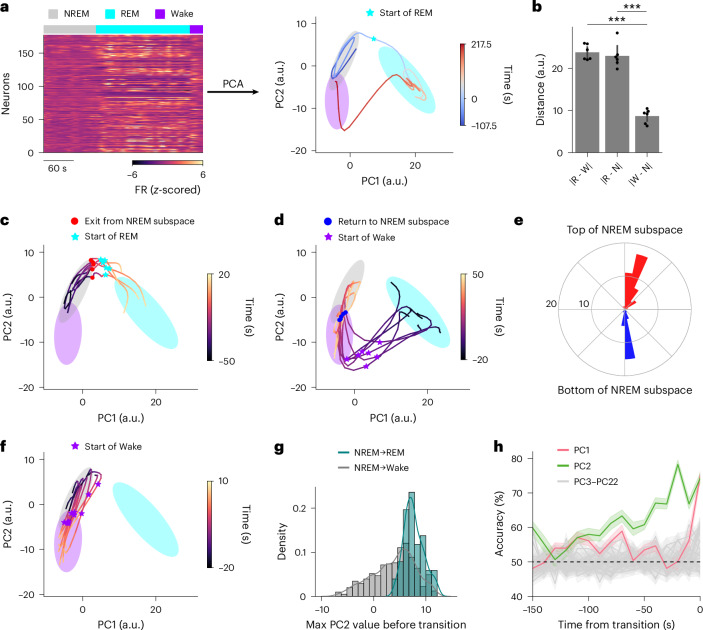
Stereotypic NREM→REM transitions in state space. **a**, Left, example population activity during a NREM→REM→Wake transition. Each row represents the color-coded firing rates (*z*-scored) of a single unit. Right, PCA was used to represent the population activity as a trajectory within the 2D state space, constructed by plotting PC2 against PC1. Time is color-coded with the REM onset at *t* = 0 s. Each ellipse captures 90% of the distribution of the population activity during REM, NREM and Wake. **b**, Distance between the NREM, REM and Wake subspaces. The distance between two subspaces is defined as the distance between the centers of the two ellipses. RM ANOVA; *P* = 1.94 × 10^−8^, *F*(2,10) = 169.41; two-sided paired *t*-tests with Holm correction, |R-N| versus |R-W|: *P* = 0.32, *T*(5) = −1.09; |R-N| versus |W-N|: *P* = 0.00018, *T*(5) = 11.40; |R-W| versus |W-N|: *P* = 6.0 × 10^−6^, *T*(5) = 24.52; *n* = 6 mice. ***P < 0.001. Dots represent individual mice; error bars denote 95% CIs. **c**, All NREM→REM trajectories within the 2D state space for one animal. Time along the trajectory is color-coded ranging from 50 s before to 20 s after the REM onset (*t* = 0 s). Red dots indicate the time points where each trajectory exited the NREM subspace. Stars depict the REM onset based on EEG/EMG annotation. **d**, REM→Wake trajectories for one animal. Time along the trajectory is color-coded ranging from 20 s before to 50 s after the REM offset (*t* = 0 s). The blue dots indicate the time points where the trajectories returned to the NREM subspace (within 50 s after the REM offset). Purple stars indicate the onset of the wake episode based on EEG/EMG annotation. **e**, Polar histogram showing the angles at which trajectories left (red) or returned (blue) to the NREM subspace relative to the principal axis of the NREM subspace ellipse. The histogram comprises the transitions from all animals. Two-sided Welch’s *t*-test, *P* = 4.83 × 10^−49^, *T*(69.50) = −39.08; *n* = 26 returns, *n* = 47 exits. **f**, NREM→Wake trajectories ranging from 20 s before to 10 s after the transition for one animal. For better visibility only every third NREM→Wake transition is shown. Purple stars indicate the Wake onset. **g**, Histogram of maximum PC2 values before a NREM→REM or NREM→Wake transition. For each NREM→REM or NREM→Wake transition from six mice, we determined the maximum PC2 value within the 30-s interval before the transition. Histograms are overlaid with kernel density estimate (KDE) plots. Two-sided Welch’s *t*-test, *P* = 2.20 × 10^−19^, *T*(112.85) = 10.92; *n* = 47 NREM→REM and *n* = 377 NREM→Wake transitions. **h**, Linear discriminant analysis (LDA) to evaluate how well a NREM→REM versus NREM→Wake transition can be predicted from the preceding activity of each individual PC (PC1–PC22). For each PC, we identified the time point *t*_sig_ from when the LDA prediction consistently exceeded chance level until the transition at *t* = 0 s (Methods). One-sided *t*-tests with Bonferroni correction; PC1: *P* = 4.98 × 10^−15^, *t*_sig_ = 0 s; PC2: *P* < 0.0038, *t*_sig_ = −110 s; *n* = 25 bootstrap iterations (see Supplementary Table 1 for results for PC3–PC22). The dashed line indicates 50% chance level. Shadings indicate the s.e.m. Source data

For further analysis, we projected the population activity into the two-dimensional (2D) state space, constructed by plotting PC2 against PC1 (Fig. 2a and Methods). A dot in the state space represents for a given time point the high-dimensional population activity reduced to a 2D vector. Connecting consecutive points in the state space results in trajectories describing the evolution of the population activity throughout time (Fig. 2a and Supplementary Video 1). For each brain state (NREM, REM sleep or Wake), as classified by EEG/EMG annotation, we determined the region (subspace) capturing 90% of the distribution of the 2D population activity within this state (Fig. 2a and Methods). The REM subspace was clearly separated from the other two subspaces, while the NREM and Wake subspaces partially overlapped (Fig. 2a,b), a structure preserved across all recorded animals (Extended Data Fig. 2).

**Extended Data Fig. 3:**
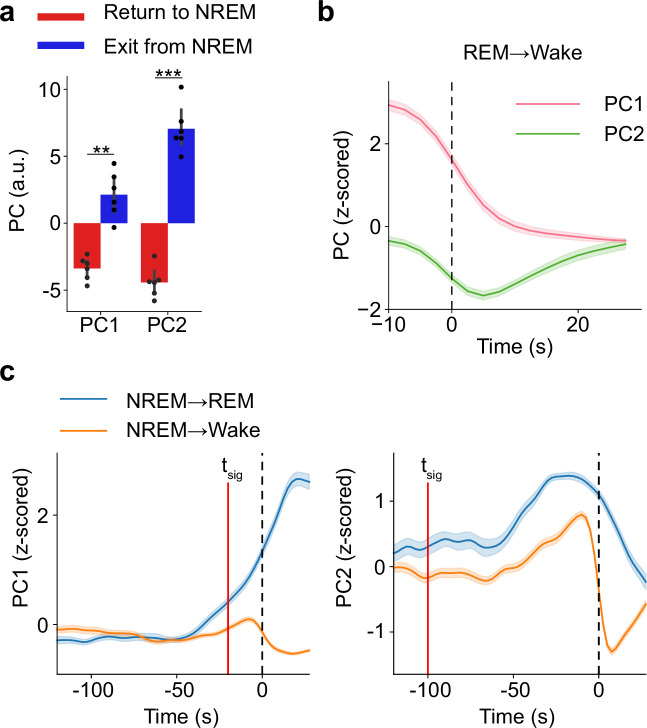
PC values at exit and return to NREM subspace, and PC activity prior to spontaneous and laser-induced REM sleep. (**a**) PC1 and PC2 values at exit and return to the NREM subspace. Two-way repeated-measures (RM) ANOVA with PC and condition (Exit vs Return) as within factors; interaction, P = 0.00033, F(1,5) = 75.60; condition, P = 4.65×10^−5^, F(1,5) = 171.28; two-sided paired t-tests with Bonferroni correction; PC1: Exit vs Return, P = 4.45e-03, T(5) = 5.76; PC2: P = 1.95×10^−6^, T(5) = 28.62. Bars, mean; error bars, 95% CIs; dots, individual mice; n = 6 mice. **P < 0.01, ***P < 0.001. (**b**) Time course of PC1 and PC2 (z-scored) relative to REM→Wake transitions (at t = 0 s). PC2 values significantly differed from 0 at t = 0 s; two-sided one-sample t-test; P = 5.41×10^−17^; T(44) = -13.28; n = 45 REM→Wake transitions from 6 mice. Shadings, s.e.m. (**c**) Time course of PC1 and PC2 (z-scored) relative to NREM→REM and NREM→Wake transitions (at t = 0 s). The red lines indicate the time point t_sig_ from when on PC1 or PC2 significantly differed prior to NREM→REM and NREM→Wake transitions. Two-sided paired t-tests with Bonferroni correction; PC1: P < 0.000045, t_sig_ = -20 s; PC2: P < 0.048, t_sig_ = -100 s; n = 47 NREM→REM, n = 190 NREM→Wake transitions. Shadings, s.e.m. See Supplementary Table 1 for full statistical details. Source data

Next, we analyzed within the state space, whether the population activity shows a common activity motif characteristic for NREM → REM transitions. During each NREM→REM transition, the trajectory of the population activity first rose along the PC2 axis toward the top of the NREM subspace, before turning into the REM subspace (Fig. 2c). Across all mice, the NREM→REM trajectories consistently exited the NREM subspace near its top (Fig. 2e and Methods). Consequently, at the exit point from NREM sleep, PC2 exhibited a large positive value, while PC1 had a relatively small positive value (Extended Data Fig. 3a). These findings demonstrate that an increase of PC2 in the population activity consistently precedes transitions to REM sleep in a stereotypic manner. In contrast, as the mouse transitioned out of REM sleep, PC2 reached negative values (Extended Data Fig. 3b), and the population activity returned to the NREM subspace at its bottom (Fig. 2d,e), where both PC1 and PC2 held negative values (Extended Data Fig. 3a). Hence, the population activity follows distinct asymmetric trajectories as the brain state switches into and out of REM sleep.

Compared with NREM→REM transitions, trajectories before NREM→Wake transitions also showed an increase along PC2, although, on average, they did not reach PC2 values as high as those observed for NREM→REM trajectories (Fig. 2f and Extended Data Fig. 3c). For direct comparison, we determined for each trajectory before a NREM→REM or NREM→Wake transition the preceding maximum PC2 value (Methods). For NREM→REM transitions, the distribution was clearly skewed toward positive PC2 values, while for NREM→Wake transitions the maximum PC2 values spanned a wide range (Fig. 2g), supporting that a large preceding PC2 value is particularly crucial for transitions to REM sleep.

Given the differences in the population activity before NREM→REM and NREM→Wake transitions, we wondered to what extent it is possible to predict whether the mouse will transition to REM sleep or wakefulness based on the preceding activity, and which of the PCs is most informative of the transition. We used a linear classifier to predict the upcoming transition to REM sleep or wakefulness using the preceding activity of each of the first 22 PCs individually (Methods). Strikingly, we found that PC2 alone consistently allowed for above chance-level predictions starting 110 s before the transition (Fig. 2h). In contrast, PC1’s predictive power was limited to the point of transition (*t* = 0 s) and, similarly, PC3 through PC22 did not allow for reliable predictions for more than 10 s before the transition (Fig. 2h and Supplementary Table 1). Thus, among the 22 PCs capturing the shared population variance, PC2 was by far the most informative of NREM→REM transitions.

### Antagonistic functional interactions along PC2

**Fig. 3:**
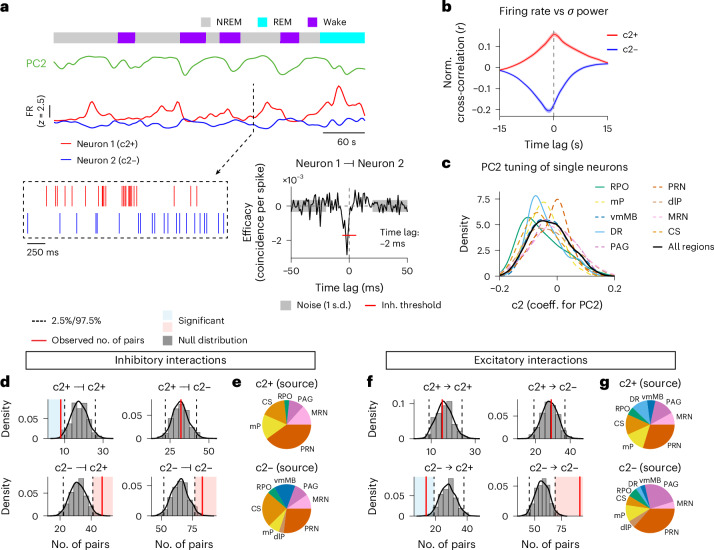
Antagonistic functional interactions along PC2. **a**, Example recording of two neurons with positive (c2+) and negative (c2−) PC2 correlation. Shown are hypnogram, PC2 and the firing rates (2.5-s bins) of both neurons. The dashed box shows the spiking activity of each neuron (1-ms bins) on an expanded timescale (each tick represents the time point of a spike). Bottom right, example of jitter-corrected CCG for a pair of neurons with inhibitory coupling. In this example, neuron 2 inhibits neuron 1 (neuron 2 ⊣ neuron 1). The red line indicates the significance threshold for inhibitory coupling. Gray regions represent ±1 s.d. of the baseline noise in the CCG side flanks (Methods). **b**, Cross-correlation between firing rates of neurons with positive (c2+) or negative (c2−) PC2 tuning and EEG *σ* power. Two-sided one-sample *t*-test (Bonferroni-corrected) to determine whether the cross-correlation peak value differs significantly from 0; c2−: *P* = 4.52 × 10^−^^94^, *T*(684) = −24.28; c2+: *P* = 2.23 × 10^−56^, *T*(345) = 19.27. Two-sided one-sample *t*-test (Bonferroni-corrected) to determine whether time lag differs significantly from 0; c2−: *P* = 3.78 × 10^−5^, *T*(684) = −4.31; mean lag = −0.35 ± 0.08 s; c2+: *P* = 0.00036, *T*(345) = 3.79; mean lag = 0.48 ± 0.13 s; c2−, *n* = 685; c2+, *n* = 346 neurons. Shadings indicate the s.e.m. **c**, Distribution of c2 coefficient across brain regions. For each neuron, c2 quantifies the strength and direction (positive or negative) of its correlation with PC2, respectively. The general distribution of c2 across all areas is shown in black. Distributions are represented as KDE plots. Solid lines represent distributions for areas whose mean significantly differed from the general c2 distribution. Two-sided Mann–Whitney *U*-test with Bonferroni correction; c2: DR, *P* = 0.015, *U* = 64847; RPO, *P* = 0.0046, *U* = 30071.5; all units, *n* = 1,160; DR, *n* = 134; PRN, *n* = 155; RPO, *n* = 69 units. **d**, Observed and expected numbers of inhibitory pairwise connections. To test whether certain pairs of inhibitory connections between c2+ and c2− neurons are overrepresented or underrepresented, we compared the observed number of connections for all four pair types to null distributions generated by randomly pairing neurons within mice. Each panel shows the null distribution (gray histogram) and KDE (black line) for one of the four possible pair types. Red vertical lines indicate the observed number of pairs; dashed black lines denote the 2.5th and 97.5th percentiles of the null distribution. Blue and red shaded regions indicate statistically significant deviations below and above chance level, respectively. We identified 186 (0.28%) inhibitory connections from a total of *n* = 67,296 pairs, recorded in 24 mice. Permutation test with Bonferroni correction; c2+ ⊣ c2+, *P* = 0.04; c2+ ⊣ c2−, *P* = 1.0; c2− ⊣ c2+, *P* = 0.008; c2− ⊣ c2−, *P* = 0.024. **e**, Distribution of inhibitory source neurons across regions. See Supplementary Table 3 for detailed counts. **f**, Observed and expected numbers of excitatory pairwise connections. We identified 172 (0.26%) excitatory connections from a total of *n* = 67,296 pairs, recorded in 24 mice. Permutation test; c2+ → c2+, *P* = 1.0; c2+ → c2−, *P* = 1.0; c2− → c2+, *P* = 0.032; c2− → c2−, *P* = 0.0. **g**, Distribution of excitatory source neurons across regions (see also Supplementary Table 3). Source data

Our analysis of the population activity in the midbrain and pons identified PC2 as a key dimension linked to the propensity to enter REM sleep. Next, we examined whether this activity component was localized to specific brain areas or broadly distributed across regions. To examine this, we computed a coefficient for each neuron (denoted as c2), quantifying its correlation with PC2 and determined its distribution across the recorded brain areas. Given the close relationship between PC2 and the *σ* power ISOs (Fig. 1h), the c2 coefficient reflects both the strength and direction of a neuron’s coupling to this infraslow rhythm: a positive c2 value (c2+; Fig. 3a) implies a positive correlation with the infraslow rhythm, while a negative c2 value (c2−) indicates a negative correlation (Fig. 3a), as shown by cross-correlating the firing rates of the c2+ and c2− neurons with the *σ* power (Fig. 3b). Interestingly, the activity of c2− neurons led on average the *σ* power during NREM sleep, as reflected in a time lag of −0.35 s ± 0.081 s in the cross-correlation (Fig. 3b and Supplementary Table 1), while c2+ neurons followed the *σ* power, suggesting that c2− neurons may play a role in modulating EEG infraslow fluctuations. Across regions, the distribution of c2 coefficients was similar, encompassing both neurons positively (c2 > 0) and negatively correlated with PC2 (c2 < 0; Fig. 3a,c). Only the DR and RPO exhibited a slight shift toward negative c2 values (Fig. 3c), demonstrating the presence of neurons negatively correlated with the ISOs in the *σ* power, as described for serotonergic neurons in the DR^12,14^. In sum, these findings suggest that PC2 captures a population-wide component that is broadly shared across neurons in the recorded midbrain and pontine regions.

**Extended Data Fig. 4:**
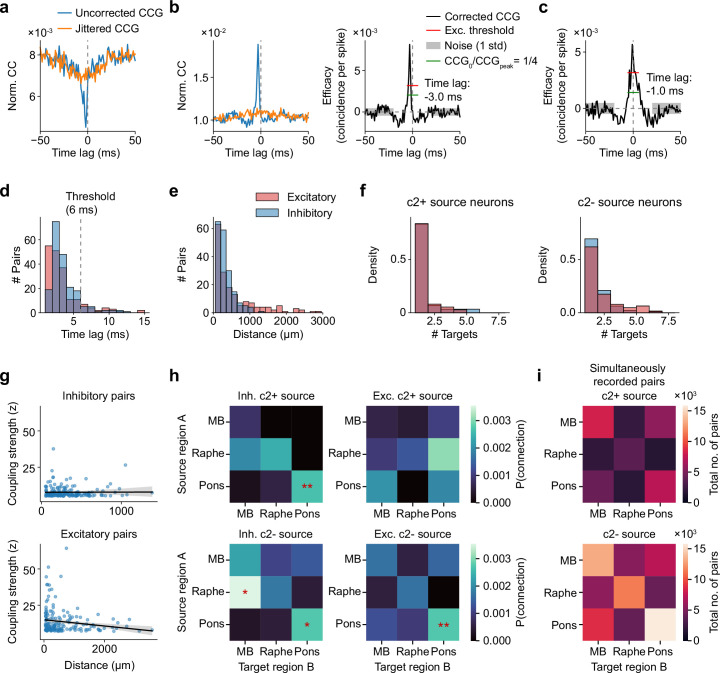
Analysis of functionally connected neuron pairs. (**a**) Raw (uncorrected) cross-correlogram (CCG) and jittered CCG for an example neuron pair with inhibitory coupling (same pair as in Fig. 3a). Subtracting the jittered CCG from the uncorrected CCG yields the jitter-corrected CCG (**Methods**). (**b**) Left, uncorrected and jittered CCG for an example pair of neurons with excitatory coupling. Right, jitter-corrected CCG. The red line indicates the threshold that the CCG peak must exceed to be classified as significant. The gray regions denote ±1 standard deviation of the baseline noise estimated from the side flanks of the CCG. To be classified as a direct excitatory connection from neuron 1 to neuron 2, the CCG value at t = 0 s (CCG_0_) had to return from its peak at a non-zero lag time (CCG_peak_) to a value below the green line, corresponding to CCG_0_ / CCG_peak_ = 1/4 (**Methods**). (**c**) Jitter-corrected CCG for an example neuron pair likely receiving common input. The CCG showed a non-zero-lag peak but a high value at t = 0 s (CCG_0_/CCG_peak_ > 1/4); this pair was therefore excluded. (**d**) Histogram over absolute time lags of positive (‘excitatory’) or negative (‘inhibitory’) peaks in the CCGs (n = 188 pairs with positive peak in the CCG and n = 199 pairs with negative peak from a total of 67,296 simultaneously recorded pairs from 24 mice). For further analysis, we only considered pairs of neurons with the CCG peak within ±6 ms (dashed vertical line; **Methods**). (**e**) Histogram over the distance between pairs of neurons with excitatory or inhibitory coupling. The distances for excitatory connections were on average larger than for inhibitory connections. Two-sided Mann-Whitney U test; P = 0.012, U = 18460.5; n = 172 excitatory and n = 186 inhibitory connections recorded in 24 mice. (**f**) Left, histograms of the number of target neurons for excitatory and inhibitory c2+ source neurons. Right, histograms of the target neuron numbers for excitatory and inhibitory c2- source neurons (15 of the source neurons were not significantly correlated with PC2). Two-sided Mann-Whitney U test; c2+ vs c2-: P = 0.014, U = 4420; c2+ , n = 67, c2-; n = 158 source neurons; excitatory vs inhibitory: P = 0.35, U = 7500; excitatory, n = 106; inhibitory, n = 134 source neurons. (**g**) Top, correlation between the z-scored peak in the CCG (measure of coupling strength) and inter-neuronal distance for excitatory connections. Bottom, correlation between negative CCG peaks and inter-neuronal distance for inhibitory connections. Linear regression; inhibitory: P = 0.93, r2 = 4.45×10^−5^; n = 186 pairs; excitatory: P = 0.031, r2 = 0.027; n = 172 pairs. Dots, individual pairs; lines, linear regression fits indicating the mean slope; shadings, 95% CIs around fitted mean. (**h**) Top, probability for each region pair A→B that a c2+ source neuron in region A inhibits (left) or excites (right) a target neuron in region B. Connection probability was computed as the number of connected pairs divided by the total number of simultaneously recorded neuron pairs between regions A and B, with the source neuron in A being c2+ (i top). Bottom, same analysis for c2- source neurons. One-sided exact binomial test with Benjamini-Hochberg false discovery rate (BH-FDR) correction; inh. c2+ source: Pons→Pons, P = 6.15×10^−3^, n = 7,228 pairs; inh. c2-: Raphe→MB, P = 0.027, n = 5,654 pairs; Pons→Pons, P = 0.027, n = 15,790 pairs; exc. c2-: Pons→Pons, P = 3.03×10^−3^, n = 15,790 pairs. *P < 0.05, **P < 0.01. See Supplementary Table 1 for full statistical details. (**i**) Total number of simultaneously recorded neurons for each region pair A→B (separated by more than 50 µm), for which the neuron in A had positive (top, c2+ source) or negative PC2 tuning (bottom, c2- source). c2-, n = 75,822; c2+ , n = 36,862 pairs. Source data

Given the existence of populations with opposing PC2 tuning, we next asked whether the c2− and c2+ neurons interact antagonistically at the synaptic level. To address this, we evaluated the functional connectivity between each pair of simultaneously recorded neurons using jitter-corrected spike cross-correlograms (CCGs; Methods)^44–46^. For the CCG calculation, spike trains were binned at 1-ms resolution, and jitter correction was applied to remove slow-timescale correlations and to isolate potential fast synaptic interactions on the millisecond scale (Fig. 3a and Methods). A sharp peak in the CCG identifies a putative synaptic interaction between the two neurons (see examples for inhibitory connection in Fig. 3a and Extended Data Fig. 4a and excitatory connection in Extended Data Fig. 4b). We restricted our analysis to neuron pairs with a peak in the CCG within ±6 ms to reduce the likelihood of capturing multisynaptic connections (Extended Data Fig. 4d)^44^. The time lag of the peak indicates the directionality of the coupling (that is, which neuron is the source or target), while the sign of the peak distinguishes excitatory (positive peak; Extended Data Fig. 4b) from inhibitory (negative peak; Fig. 3a) interactions. In 24 mice, we identified from a total of 67,296 possible pairs 172 (0.26%) excitatory and 186 (0.28%) inhibitory (0.51% ± 0.06% pairs per mouse) connections^44^. Excitatory connections spanned overall longer distances than inhibitory ones (Extended Data Fig. 4e). A total of 27.9% of source neurons formed connections with more than one postsynaptic partner, with an average of 1.49 ± 0.06 target neurons. Across the population, c2− source neurons exhibited a higher degree of connectivity than c2+ source neurons (Extended Data Fig. 4f). The excitatory coupling strength decayed with interneuronal distance, while inhibitory coupling showed no significant distance dependence (Extended Data Fig. 4g).

Based on the sign of their PC2 coefficients, we classified neuron pairs into four categories (c2+ ~ c2+, c2+ ~ c2−, c2− ~ c2+, and c2− ~ c2−). For each category, we counted the number of putative synaptic connections across all recorded areas and compared it with a null distribution generated by randomly pairing neurons out of the pool of all recorded neurons (Methods). This analysis allowed us to determine whether any pair type is significantly overrepresented or underrepresented relative to chance. Interestingly, we found that inhibitory connections from c2− to c2+ neurons (c2− ⊣ c2+) are more frequent than expected (Fig. 3d). Second, we observed that inhibitory connections among c2− neurons were overrepresented (Fig. 3d; c2− ⊣ c2−), whereas c2+ ⊣ c2+ connections were less frequent than expected. Performing the same analysis for putative excitatory connections revealed strong excitation among the population of c2− neurons (Fig. 3f; c2− → c2−), whereas connections from c2− to c2+ neurons were underrepresented (Fig. 3f; c2− → c2+). These connectivity motifs indicate that strong excitation within the c2− population is matched by self-inhibition. Importantly, the asymmetry in the number of inhibitory connections between c2− and c2+ neurons suggests that neurons with negative PC2 tuning exert strong inhibitory control over c2+ neurons across the recorded midbrain and pontine areas.

Next, we examined how putative inhibitory (that is, source neurons in CCGs with negative peaks; Fig. 3a) and excitatory (positive peaks; Extended Data Fig. 4b) source neurons are distributed across regions. The majority of inhibitory c2+ and c2− source neurons were located in two pontine regions (Fig. 3e), the PRN and CS, accounting for 56.7% (17/30) of c2+ and 50.5% (47/93) of c2− inhibitory neurons. Most putative excitatory source neurons were located in the PRN in the pons and PAG in the midbrain (43.2% (16/37) of c2+ and 60.3% (38/60) of c2− neurons; Fig. 3g). Next, to examine whether c2+ and c2− neurons preferentially formed connections within or between brain regions, we first grouped brain areas into pons (PRN, dlP, mP), midbrain (PAG, MRN, vmMB), and raphe nuclei (DR, CS and RPO), and computed connection probabilities for each region pair. We then identified region pairs for which the probability that a pair of neurons was connected was significantly enhanced relative to the overall baseline connection probability (Methods and Extended Data Fig. 4h,i). In addition to the large number of source neurons in the pons (Fig. 3e,g), we observed an enriched local connectivity among pontine neurons (Extended Data Fig. 4h). Inhibitory c2+ and c2− neurons and excitatory c2+ neurons showed significantly increased connection probabilities with other pontine neurons. Together, these findings indicate that the pons forms a densely interconnected network along the PC2 axis, characterized by enriched local inhibitory connectivity.

### Antagonistic infraslow and ultradian activity dynamics

**Fig. 4:**
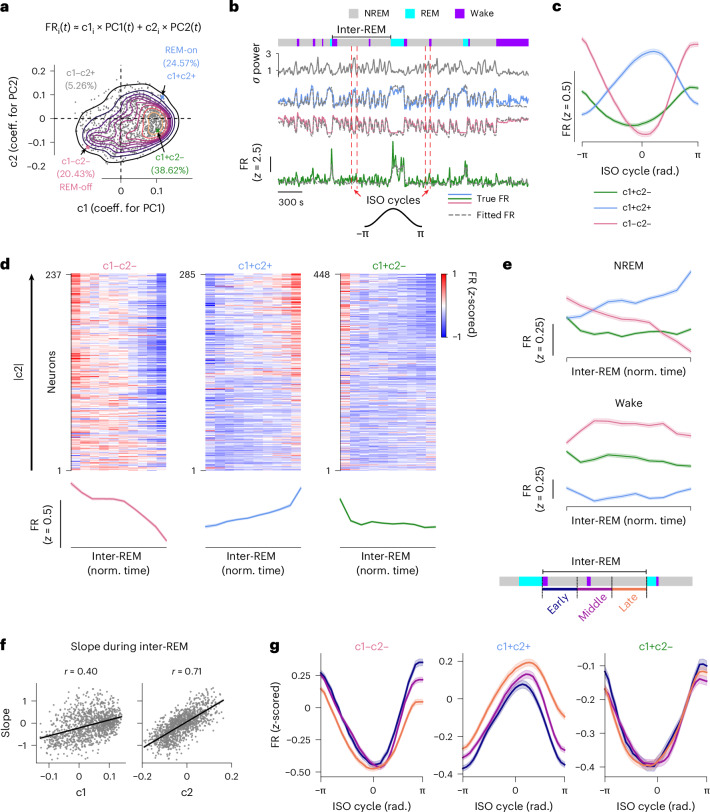
Antagonistic infraslow and ultradian dynamics across brain areas. **a**, The firing rates of each unit can be approximated by a weighted sum of PC1 and PC2. The scatterplot shows for each unit the two coefficients c1 and c2 for PC1 and PC2 (see formula). **b**, Firing rates and fit using only PC1 and PC2 for three example neurons, indicated by the arrows and colored crosses in **a**. The red dashed lines indicate two ISO cycles. **c**, Activity of different neuron subclasses throughout the ISO cycle. The duration of each ISO cycle was normalized in time and the *z*-scored activity of each unit was averaged across multiple ISO cycles. c1−c2−, *n* = 237; c1+c2+, *n* = 285; c1+c2−, *n* = 448 units. Shadings indicate the s.e.m. **d**, Activity of different neuron subclasses during inter-REM interval. Top, heat maps depicting for each subclass the mean activity of individual neurons (rows) throughout the inter-REM interval, with units sorted by their absolute c2 value (|c2|). For time normalization, each inter-REM interval was divided in ten equally long bins, and the *z*-scored activity of each unit was averaged for each bin across multiple inter-REM intervals. Bottom, average activity for each subclass. c1−c2−, *n* = 237; c1+c2+, *n* = 285; c1+c2−, *n* = 448 units. Shadings indicate the s.e.m. **e**, Firing rates of different neuron subclasses during NREM (top) and wake (bottom) states across the inter-REM interval. Each inter-REM interval was divided into ten bins, and the NREM or wake activity of each unit was averaged within each bin across multiple inter-REM intervals. c1−c2−, *n* = 237; c1+c2+, *n* = 285; c1+c2−, *n* = 448 units. Shadings indicate the s.e.m. **f**, Correlation of c1 and c2 with slope during inter-REM. For each unit, we determined the slope at which its firing rates changed throughout inter-REM (Methods) and plotted it against its c1 and c2 value. Dots represent single units. Lines depict regression fits, indicating the mean slope; shadings indicate the 95% CIs around the fitted mean. Linear regression; c1 versus slope: *r* = 0.40, *P* = 0.0; c2 versus slope: *r* = 0.71, *P* = 0.0; *n* = 1,160 units. **g**, Top, schematic illustrating partitioning of inter-REM into early, middle and late sections. Bottom, activity of different neuron subclasses across ISO cycles during early, middle and late inter-REM sections. c1−c2−, *n* = 237; c1+c2+, *n* = 285; c1+c2−, *n* = 448 units. Shadings indicate the s.e.m. Source data

**Extended Data Fig. 5:**
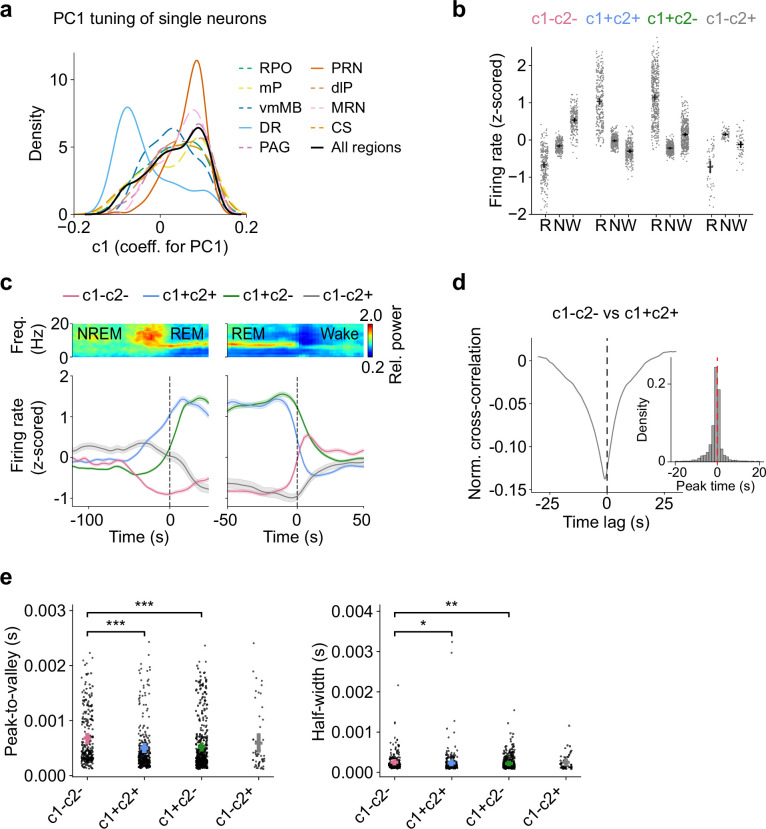
Activity, firing rate correlations, and waveform properties of different neuron subclasses. (**a**) Distribution of c1 coefficient across brain regions. For each neuron, c1 quantifies the strength and direction (positive or negative) of its correlation with PC1. The general distribution for c1 across all areas is shown in black. Distributions are represented as KDE plots. Solid lines, distributions for areas whose mean significantly differed from the general c1 distribution (black). Two-sided Mann-Whitney U test with Bonferroni correction, c1: DR, P = 1.04×10^−22^, U = 36649; PRN, P = 4.03×10^−5^, U = 110272; all units, n = 1160; DR, n = 134; PRN, n = 155 units. (**b**) Average firing rates of c1-c2-, c1+c2+ , c1+c2-, and c1-c2+ neurons for each brain state. c1-c2-: RM ANOVA, P = 3.38×10^−114^, F(2, 472) = 677.04; two-sided paired t-tests with Bonferroni correction, N vs R: P = 3.90×10^−39^, T(236) = 16.03; N vs W: P = 5.49×10^−73^, T(236) = -26.77; R vs W: P = 2.13×10^−83^, T(236) = -30.51; c1+c2+ : RM ANOVA, P = 2.15×10^−108^, F(2, 568) = 753.54; two-sided paired t-tests, N vs R: P = 4.28×10^−71^, T(284) = -24.37; N vs W: P = 7.43×10^−34^, T(284) = 14.030; R vs W: P = 1.44×10^−97^, T(284) = 32.62; c1+c2-: RM ANOVA, P = 2.68×10^−145^, F(2, 894) = 997.41; two-sided paired t-tests, N vs R: P = 5.86×10^−161^, T(447) = -43.14; N vs W: P = 3.58×10^−62^, T(447) = -19.70; R vs W: P = 2.04×10^−84^, T(447) = 24.55; c1-c2+ : RM ANOVA, P = 7.27×10^−15^, F(2,120) = 69.36; two-sided paired t-tests, N vs R: P = 2.31×10^−16^, T(60) = 11.52; N vs W: P = 1.07×10^−6^, T(60) = 5.72; R vs W: P = 1.46×10^−7^, T(60) = -6.24; c1-c2-, n = 237; c1+c2+, n = 285; c1+c2-, n = 448; c1-c2+, n = 61 units. Dots, individual units; error bars, 95% CIs. R, REM; N, NREM; W - Wake. (**c**) Activity of c1-c2-, c1+c2+, c1+c2-, and c1-c2+ neurons at NREM→REM and REM→Wake transitions. Time point t_sig_ from when on the activity consistently differed from baseline (two-sided t-tests with Bonferroni correction; **Methods**), c1-c2-: t_sig_ = -55 s, P < 0.00047; c1+c2+: t_sig_ = -45 s, P < 0.0047; c1+c2-: t_sig_ = -5 s, P < 0.000049; c1-c2-, n = 237; c1+c2+, n = 285; c1+c2-, n = 448; c1-c2+ , n = 61 units. Lines, mean across units; shadings, 95% CIs. See Supplementary Table 1 for full statistical details. (**d**) Cross-correlation between c1-c2- and c1+c2+ neurons during NREM sleep (**Methods**). Shadings, 95% CIs. Inset, histogram of the time lags in the cross-correlograms of negatively correlated neurons. The average time lag was -1.22 s ± 0.08 s, indicating that the activity of c1-c2- neurons leads that of c1+c2+ neurons; two-sided one sample t-test; P = 3.56×10^−52^, T(7878) = -15.31; n = 7879 pairs from 6 mice. (**e**) Peak-to-valley interval and half-width of the spike waveforms for the different neuron subclasses. Peak-to-valley: Kruskal-Wallis test; P = 3.80×10^−8^, H(3) = 37.39; two-sided Mann-Whitney U test (Bonferroni-corrected), c1+c2- vs c1-c2-: P = 9.37×10^−8^, U = 39156.5; c1+c2+ vs c1-c2-: P = 5.94×10^−7^, U = 24629.5; c1-c2+ vs c1-c2-: P = 0.12, U = 5837.5; half-width: Kruskal-Wallis test; P = 5.42×10^−3^, H(3) = 12.67; two-sided Mann-Whitney U test (Bonferroni-corrected), c1+c2- vs c1-c2-: P = 7.46×10^−3^, U = 45134.0; c1+c2+ vs c1-c2-: P = 0.01, U = 28390.5; c1+c2- vs c1-c2-: P = 0.0075, U = 45134; c1-c2-, n = 237; c1+c2+ , n = 285; c1+c2-, n = 448; c1-c2+, n = 61 units. *P < 0.05, **P < 0.01, ***P < 0.001. Horizontal lines, mean; error bars, 95% CIs. See Supplementary Table 1 for full statistical details. Source data

Given the low dimensionality of the population activity, the firing rates of each neuron can be well approximated as a weighted combination of the first two PCs (Fig. 4a,b). The coefficient for PC1 (c1) captures how strongly a neuron is activated (c1 > 0) or inhibited (c1 < 0) during REM sleep, while the c2 coefficient, as mentioned above, reflects its coupling with the EEG ISOs. Like the c2 coefficient (Fig. 3c), c1 values showed a similar distribution across brain areas, except for the DR and PRN (Extended Data Fig. 5a). In the DR, c1 values were negatively skewed, consistent with numerous neurons minimally active during REM sleep^12,28,29,33^, whereas in the PRN they were biased positively, reflecting abundant REM sleep-activated neurons, in line with previous recordings in the dorsal pons^47–49^.

By plotting c1 against c2, the firing rates of each neuron can be represented as a point in a 2D scatterplot (Fig. 4a). Most neurons with a negative c1 coefficient also had a negative c2 coefficient. Because of their negative correlation with PC2, activation of these neurons in the lower-left quadrant (c1−c2− neurons; pink neuron in Fig. 4b) is expected to drive the population activity toward negative PC2 values, thereby preventing transitions to REM sleep; therefore, we refer to them as REM-off neurons. REM sleep-activated neurons (c1 > 0) comprise two large subpopulations with opposing PC2 tuning: c1+c2− neurons (negative PC2 correlation; green neuron in Fig. 4b) and c1+c2+ neurons (positive PC2 correlation; blue neuron in Fig. 4b). According to our geometric analysis (Fig. 2), only the positively tuned c1+c2+ neurons (upper-right quadrant) are expected to function as REM-on neurons, pushing the population activity toward the top of the NREM subspace.

The putative REM-on (c1+c2+) and REM-off (c1−c2−) neurons comprised comparable proportions (Fig. 4a) and, consistent with their opposing PC tuning, showed opposing state-dependent mean firing rates (Extended Data Fig. 5b). At NREM→REM transitions, the firing rates of c1+c2+ and c1−c2− neurons started diverging in a mirror-symmetric manner about 50 s before the transition (Extended Data Fig. 5c and Supplementary Table 1), consistent with their predicted antagonistic roles in gating REM sleep. At the REM→Wake transition, c1−c2− neurons rapidly increased their firing rates, while the activity of c1+c2+ neurons abruptly dropped (Extended Data Fig. 5c). As expected from their opposing PC1 and PC2 tuning, cross-correlating the firing rates of individual c1−c2− and c1+c2+ neurons during NREM sleep revealed a negative correlation (Extended Data Fig. 5d). To test for directionality, we examined the time lag distribution of negatively correlated pairs. Peak times were skewed toward negative values (−1.22 s ± 0.08 s; Extended Data Fig. 5d), indicating that REM-off neurons overall lead the REM-on neuron activity.

Most neurons fell into the c1+c2− subclass (Fig. 4a). Like REM-on (c1+c2+) neurons, this subpopulation was most active during REM sleep (Extended Data Fig. 5b); however, their negative PC2 tuning predicts that they suppress NREM→REM transitions. But, given that c1+c2− neurons only showed a comparably weak reduction in their firing rates before NREM→REM transitions, followed by an increase (Extended Data Fig. 5c), their activity may contribute less to suppressing REM sleep than that of c1−c2− neurons. The smallest fraction of neurons (5.26%) fell into the c1−c2+ quadrant (Fig. 4a), characterized by maximal firing rates during NREM sleep (Extended Data Fig. 5b,c). Neuron subclasses exhibited small differences in spike waveform properties, with c1−c2− neurons showing the broadest waveforms, based on peak-to-valley intervals and half-widths, among the three major subclasses (Extended Data Fig. 5e).

**Extended Data Fig. 6:**
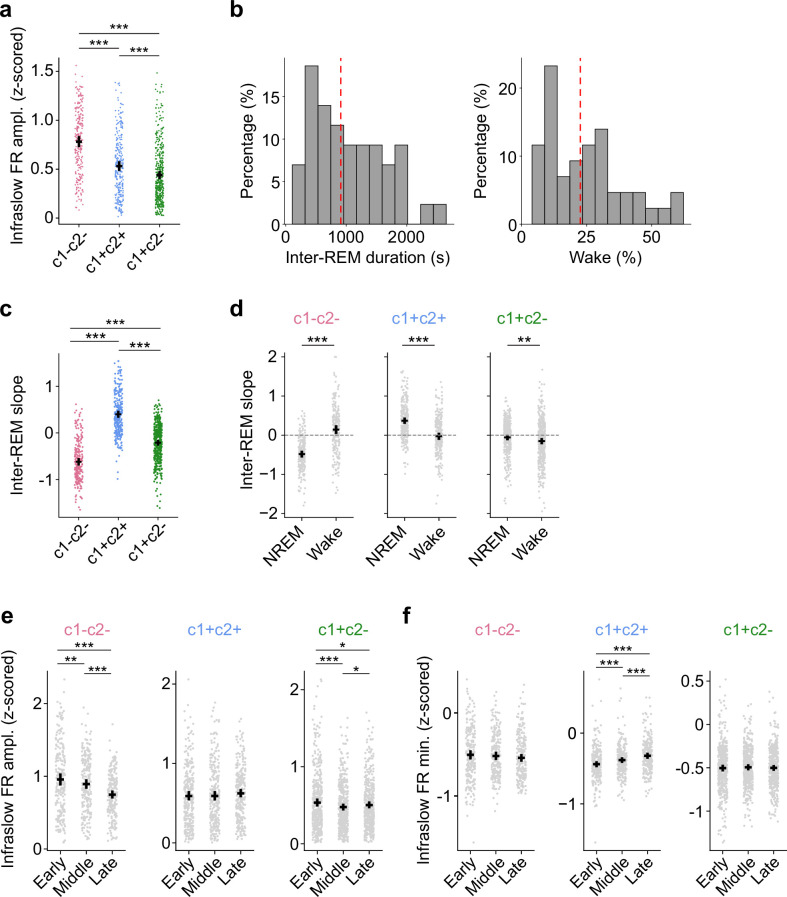
Inter-REM dynamics of different neuron subclasses. (**a**) Amplitude of infraslow activity modulation in different neuron subclasses. The infraslow firing rate amplitude for each neuron was quantified as the difference between the maximum and minimum firing rates within ISO cycles (identified using EEG σ power). FR, firing rates. Horizontal lines, mean; vertical lines, 95% CIs; dots, individual units. ANOVA; P = 5.48×10^−38^, F(2, 967) = 93.88; two-sided t-tests with Bonferroni correction; c1+c2+ vs c1-c2-: P = 2.63×10^−16^, T(487.52) = -8.63; c1+c2- vs c1-c2-: P = 1.07×10^−32^, T(422.38) = -13.11; c1+c2+ vs c1+c2-: P = 2.73×10^−4^, T(570.17) = 3.94; c1-c2-, n = 237; c1+c2+ , n = 285; c1+c2-, n = 448 units. (**b**) Left, histogram showing distribution of inter-REM interval durations. Red dashed line, median of distribution (910.0 s). Right, distribution of wake percentages during inter-REM intervals. Red dashed line, median (22.54%); n = 43 inter-REM intervals from 6 mice. (**c**) Slope at which the activity of the neurons in each subclass increased or decayed throughout inter-REM. For time normalization, each inter-REM interval was divided into 10 equally long bins. The slope at which the z-scored activity of each unit changed throughout inter-REM was fitted using linear regression. To exclude fast changes in neural activity immediately after or before REM sleep, we dropped the first and last bin of the inter-REM interval for the regression analysis (**Methods**). Horizontal lines, mean; vertical lines, 95% CIs; dots, individual neurons. ANOVA; P = 5.11×10^−128^, F(2, 967) = 402.98; two-sided t-tests with Bonferroni correction; c1+c2+ vs c1-c2-: P = 5.71×10^−98^, T(506.19) = 26.65; c1+c2+ vs c1+c2-: P = 2.01×10^−62^, T(554.41) = 19.11; c1+c2- vs c1-c2-: P = 4.48×10^−29^, T(443.44) = 12.16; c1-c2-, n = 237; c1+c2+, n = 285; c1+c2-, n = 448 units. ***P < 0.001. (**d**) Slope at which different subclasses increased or decreased during NREM or wake episodes throughout inter-REM. Each inter-REM interval was divided into 10 bins. For each bin, we determined the average wake or NREM activity, before performing linear regression analysis. The first and last bin were dropped. Horizontal lines, mean; vertical lines, 95% CIs; dots, individual neurons. Two-sided t-tests with Bonferroni correction comparing slope for NREM and wake; c1-c2-: P = 1.31×10^−38^, T(236) = 15.87; c1+c2+: P = 7.49×10^−36^, T(284) = -14.58; c1+c2-: P = 0.002, T(447) = -3.48; c1-c2-, n = 237; c1+c2+, n = 285; c1+c2-, n = 448 units. **P < 0.01, ***P < 0.001. (**e**) Amplitude of infraslow activity modulation in different neuron subclasses during early, middle, and late inter-REM sections. The infraslow firing rate amplitude was quantified as the difference between the minimum and maximum firing rates within ISO cycles. Inter-REM intervals were divided into three equally sized sections (early, middle, late). Horizontal lines, mean; vertical lines, 95% CIs; dots, individual neurons. RM ANOVA with inter-REM section as within factor; c1-c2-: P = 4.70×10^−23^, F(2, 472) = 57.44; c1+c2+: P = 0.04, F(2, 568) = 3.30; c1+c2-: P = 1.15×10^−6^, F(2, 894) = 13.89; two-sided t-tests with Bonferroni correction; c1-c2-: early vs middle: P = 0.004, T(236) = 3.25; early vs late: P = 3.31×10^−16^, T(236) = 8.95; middle vs late: P = 4.46×10^−16^, T(236) = 8.90; c1+c2+: early vs middle: P = 1, T(284) = 0.07; early vs late: P = 0.13, T(284) = 2.04; middle vs late: P = 0.13, T(284) = 2.03; c1+c2-: early vs middle: P = 2.52×10^−8^, T(447) = 5.87; early vs late: P = 0.039, T(447) = 2.50; middle vs late: P = 0.033, T(447) = 2.55; c1-c2-, n = 237; c1+c2+, n = 285; c1+c2-, n = 448 units. *P < 0.05, **P < 0.01, ***P < 0.001. (**f**) Minimum firing rates during ISO cycles within early, middle, and late inter-REM sections. Horizontal lines, mean; vertical lines, 95% CIs; dots, individual neurons. RM ANOVA with inter-REM section as within factor; c1-c2-: P = 0.055, F(2, 472) = 2.92; c1+c2+: P = 2.32×10^−18^, F(2, 568) = 43.65; c1+c2-: P = 0.56, F(2, 894) = 0.59; two-sided t-tests with Bonferroni correction; c1+c2+: early vs middle: P = 3.26×10^−5^, T(284) = 4.48; early vs late: P = 3.87×10^−15^, T(284) = 8.48; middle vs late: P = 2.28×10^−7^, T(284) = 5.52; c1-c2-, n = 237; c1+c2+, n = 285; c1+c2-, n = 448 units. ***P < 0.001. Source data

Having established antagonistic activity patterns between c1−c2− and c1+c2+ neurons at REM sleep transitions (Extended Data Fig. 5c), we next investigated whether these subpopulations also exhibit opposing dynamics on slower timescales during sleep. To compare their dynamics on the infraslow timescale, we calculated for the different neuron subclasses the average activity during single (time-normalized) ISO cycles (Fig. 4c and Methods). Consistent with their opposing PC2 tuning, c1−c2− and c1+c2+ neurons showed a strong antagonistic phase tuning, with c1−c2− neurons least active at the peak of the infraslow cycle, where c1+c2+ neurons were maximally active (Fig. 4c). Compared with these two subclasses, c1+c2− neurons exhibited the weakest modulation in their amplitude across the ISO cycle (Fig. 4c and Extended Data Fig. 6a).

REM sleep in mice recurs on an ultradian timescale of several minutes^4,5^. An ultradian signature of mammalian sleep is the positive correlation between REM sleep duration and subsequent NREM sleep^4,6,8^. Comparing sleep in head-fixed and freely moving mice, we confirmed that this relationship is preserved under head fixation (Supplementary Fig. 2d). We next asked whether the neural dynamics in the midbrain and pons also exhibit slower ultradian modulations in synchrony with the NREM–REM cycle, beyond infraslow fluctuations. We analyzed for each unit its modulation throughout the interval between consecutive REM sleep episodes (inter-REM interval; Fig. 4b). The median duration of inter-REM intervals was 910 s with a median wake content of 22.54% (Extended Data Fig. 6b). We normalized the duration of each inter-REM interval and averaged for each unit its activity over multiple intervals (Fig. 4d and Methods). Interestingly, the subpopulations of REM-off (c1−c2−) and REM-on (c1+c2+) neurons showed antagonistic ramping dynamics throughout the inter-REM interval. The firing rates of c1−c2− neurons gradually decayed, reaching their lowest level at the onset of the next REM episode, while the c1+c2+ activity slowly increased throughout inter-REM (Fig. 4d and Extended Data Fig. 6c). Given that a large PC2 value consistently precedes transitions to REM sleep (Fig. 2c,e), these diverging ramping dynamics likely reflect a gradual buildup in the propensity to enter REM sleep throughout inter-REM. Analyzing the activity within NREM and wake states separately during the inter-REM interval, we found that the ramping activity patterns of c1+c2+ and c1−c2− neurons were more pronounced during NREM sleep than during wakefulness (Fig. 4e and Extended Data Fig. 6d), suggesting that the accumulation of REM propensity occurs primarily during NREM sleep^4,6,8^. In contrast to the c1−c2− subpopulation, c1+c2− neurons showed on average only a weak decline in their firing rates throughout the inter-REM interval (Extended Data Fig. 6c), present during both NREM sleep and wake (Extended Data Fig. 6d), following an initial steep drop in their activity (Fig. 4d).

Thus, REM-off (c1−c2−) and REM-on (c1+c2+) neurons showed opposing activity dynamics across multiple timescales, from rapid state transitions (Extended Data Fig. 5c), to infraslow (Fig. 4c) and ultradian activity modulations (Fig. 4d). Next, we wondered whether the infraslow and ultradian firing rate fluctuations were related within individual neurons. To quantify the strength of the ultradian modulation, we measured for each neuron the slope at which its activity changed throughout inter-REM and found that it was positively correlated with the c1 and c2 coefficients (Fig. 4f). The correlation was particularly robust for c2, demonstrating that neurons with pronounced infraslow rhythmicity also tended to show strong ultradian modulation.

Because REM-on and REM-off neurons showed strong infraslow and ultradian modulation, we next asked whether the gradual change in their firing rates throughout inter-REM resulted from an increase in the amplitude of their infraslow rhythmicity or, instead, from shifts in their baseline activity (Fig. 4g). To address this, we quantified for the different neuron subclasses the amplitude of their infraslow modulation and their minimum activity (as a proxy for baseline activity) within ISO cycles across three consecutive sections of the inter-REM interval (early, middle and late; Fig. 4g and Methods). REM-off (c1−c2−) neurons showed a progressive decay (early > middle > late) in their infraslow amplitude from the early to the late section (Fig. 4g and Extended Data Fig. 6e). Conversely, REM-on (c1+c2+) neurons exhibited no progressive change in their amplitude across the three sections (Extended Data Fig. 6e), but their minimum activity increased steadily throughout inter-REM (early < middle < late; Fig. 4g and Extended Data Fig. 6f), consistent with a slow rise in baseline activity. The c1+c2− population displayed no significant progressive increase or decrease in either amplitude or minimum activity across inter-REM (Fig. 4g, Extended Data Fig. 6e,f and Methods). Together, these findings suggest that, as time elapses since the last REM episode and REM pressure accrues, the baseline activity of REM-on neurons gradually rises, whereas the amplitude of the infraslow modulation in REM-off neurons weakens.

### PC2 gates the impact of upstream REM-on neuron activation

**Fig. 5:**
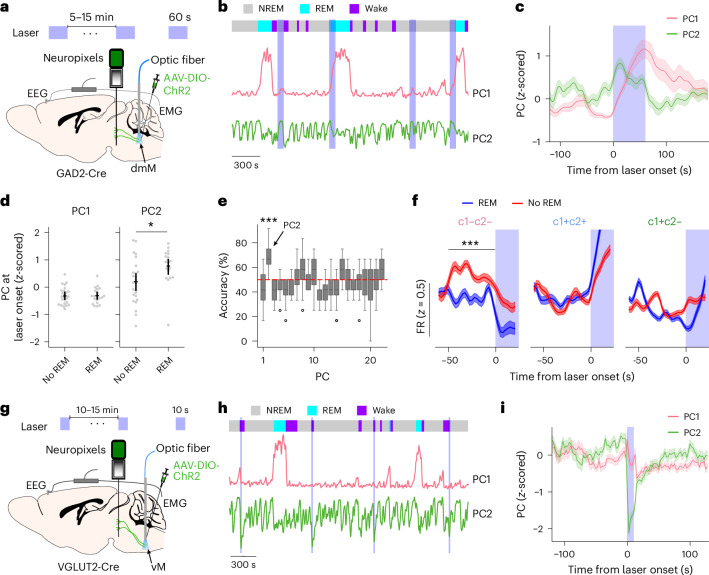
PC2 gates the impact of upstream REM-on neuron activation. **a**, Schematic illustrating Neuropixels recordings in midbrain and pons of a GAD2-Cre mouse, while optogenetically activating dmM GAD2 neurons. The laser was turned on every 5–15 min for 60 s (10 Hz). **b**, Example recording showing a hypnogram along with PC1 and PC2, while stimulating dmM GAD2 neurons. Blue shadings indicate the timing of the 60-s laser trials. **c**, Trial-averaged PC1 and PC2 activity before, during and after laser stimulation for trials where the laser onset fell on NREM (including both successful and unsuccessful trials). Two-way RM ANOVA comparing the mean value of each PC during the laser and preceding 60-s baseline interval; interaction, *P* = 0.0091, *F*(1,176) = 6.9484; two-sided paired *t*-tests (Bonferroni-corrected); baseline versus laser: PC1, *P* = 1.32 × 10^−11^, *T*(44) = 9.27; PC2, *P* = 0.0013, *T*(44) = 3.67; *n* = 45 laser trials from five mice; shadings indicate 95% CIs. **d**, PC1 and PC2 values at the onset of successful (REM) and unsuccessful (no REM) laser trials (restricted to trials in which the mouse was in NREM sleep at laser onset). Two-way ANOVA with PCs and laser outcome as between factors; interaction, *P* = 0.0028, *F*(1,86) = 9.47; two-sided *t*-tests (Bonferroni-corrected); PC1: REM versus no REM, *P* = 0.17, *T*(38.76) = 1.78; PC2: *P* = 0.020, *T*(42.64) = −2.70; *n* = 20 successful, *n* = 25 unsuccessful trials. Horizontal lines indicate the mean; error bars indicate 95% CIs. *P < 0.05. **e**, LDA to test whether a transition to REM sleep during the laser interval can be predicted from the values of PC1–PC22 at laser onset. Box plots (see Methods for definition) represent for each PC the prediction accuracy. One-sided *t*-tests with Bonferroni correction to determine whether prediction accuracy exceeds chance level; PC2: *P* = 0.00018, *T*(9) = 8.34; *n* = 10 bootstrap iterations. Dashed line indicates 50% chance level; error bars indicate 95% CIs. ***P < 0.001. **f**, Activity of c1−c2−, c1+c2+ and c1+c2− neurons relative to the onset of successful and unsuccessful laser trials (including only trials with laser onset during NREM). Mixed ANOVA to determine for each subclass (between factor) differences in the mean activity during the 50-s interval before laser onset between successful and unsuccessful trials (within factor); interaction, *P* = 2.58 × 10^−5^, *F*(3,552) = 8.14; two-sided *t*-tests with Bonferroni correction; c1−c2−: REM versus no REM, *P* = 2.82 × 10^−^^5^, *T*(132) = 4.68; c1−c2−, *n* = 133; c1+c2+, *n* = 153; c1+c2−, *n* = 246 units. See Extended Data Fig. 7j for statistical details. ***P < 0.001. Shadings indicate the s.e.m. **g**, Schematic illustrating Neuropixels recordings in midbrain and pons of a VGLUT2-Cre mouse, while optogenetically activating arousal-promoting neurons in the vM. The laser was turned on every 10–15 min for 10 s (10 Hz). **h**, Example recording showing a hypnogram along with PC1 and PC2, while stimulating vM VGLUT2 neurons. **i**, Trial-averaged PC1 and PC2 activity before, during and after laser stimulation of vM VGLUT2 neurons for trials where the laser onset fell on NREM sleep. Two-way RM ANOVA comparing the mean value of each PC during the laser and preceding 60-s baseline interval; interaction, *P* = 1.82 × 10^−9^, *F*(1,41) = 59.03; two-sided paired *t*-tests (Bonferroni-corrected); baseline versus laser: PC1, *P* = 0.47, *T*(41) = −1.21; PC2, *P* = 5.16 × 10^−23^, *T*(41) = 20.69; *n* = 42 laser trials (from five recordings in four mice); shadings indicate 95% CIs. Source data

**Extended Data Fig. 7:**
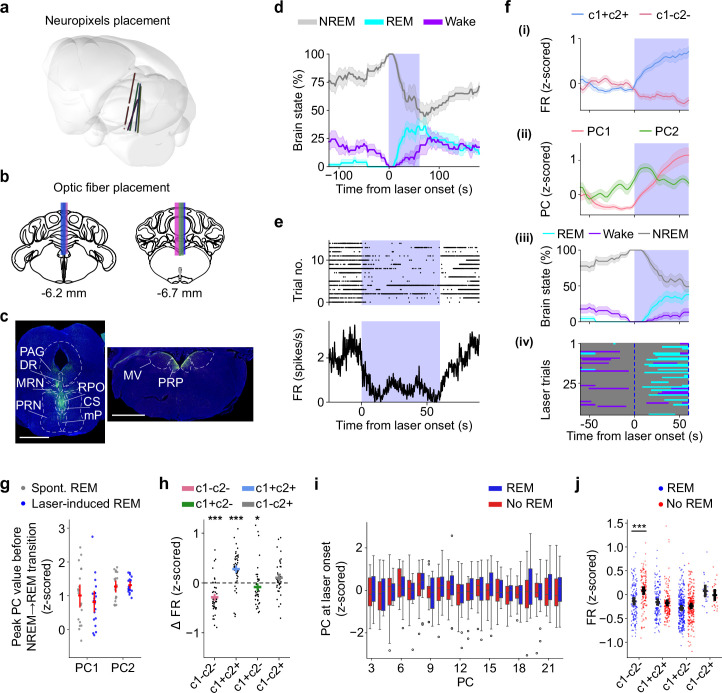
Optogenetic stimulation of dmM GAD2 neurons and its effect on midbrain/pontine population activity. (**a**) Anatomical reconstruction of electrode tracts in GAD2-Cre mice injected with AAV-DIO-ChR2-eYFP (n = 5 mice). (**b**) Location of optic fibers for optogenetic activation of dmM GAD2 neurons. Each colored bar represents the location of an optic fiber in a GAD2-Cre mouse (n = 5 mice). (**c**) Fluorescence images showing expression of ChR2-eYFP in the dmM of a GAD2-Cre mouse (right) and axonal projections of dmM GAD2 neurons in midbrain and pons (left). PRP, nucleus prepositus; MV, medial vestibular nucleus. Blue, DAPI stain. Scale bars, 1 mm. (**d**) Percentage of Wake, NREM, and REM sleep before, during, and after optogenetic stimulation of dmM GAD2 neurons, including only trials with the laser onset (t = 0 s) falling on NREM sleep. Two-way RM ANOVA comparing the mean percentage of each brain state during the 60-s laser and preceding 60-s baseline interval; interaction, P = 0.0099, F(2, 8) = 10.24; two-sided paired t-tests with Bonferroni correction; REM: P = 0.037, T(4) = -4.344; Wake: P = 0.9203, T(4) = 1.17; NREM: P = 0.14, T(4) = 2.88; n = 5 mice. Shadings, s.e.m. (**e**) Top, spike raster showing for an inhibited unit all 60-s laser trials. Bottom, average laser trial-averaged firing rates. Blue shading, laser interval. (**f**) Comparison of firing rates, PCs, brain state percentages, and single laser trials. i, Firing rates (FR, z-scored) of c1+c2+ and c1-c2- neurons. ii, Time course of PC1 and PC2. iii, Percentages of each brain state. iv, Heatmap depicting the hypnogram for each laser trial. Solid lines in i-iii show averages across laser trials (n = 45). i-iv only include trials with laser onset during NREM. Shadings, s.e.m. (**g**) Maximum PC1 and PC2 values before NREM→REM or NREM→Wake transitions. For each NREM→REM transition, we determined the peak PC1 and PC2 value within the 30-s interval before the transition. Two-way ANOVA; interaction, P = 0.21, F(1, 82) = 1.58; two-sided t-tests with Bonferroni correction; PC1: P = 0.43, T(39.24) = 1.26; PC2: P = 1, T(35.72) = -0.26; n = 22 spontaneous and n = 21 laser-induced REM episodes. Horizontal lines, mean; error bars, 95% CIs. (**h**) Laser-induced firing rate changes across neuron subclasses (including only trials with laser onset during NREM). Two-sided one-sample tests (Bonferroni-corrected) for mean firing rate changes (50 s pre- vs 5 s post-laser); c1-c2-: P = 1.14×10^−5^, W = 130.00; c1+c2+: P = 3.20×10^−5^, W = 145.00; c1+c2-: P = 0.0255, W = 279.00; c1-c2+: P = 0.2738, T(38) = 1.88; n = 45 laser trials. Horizontal lines, mean; error bars, 95% CIs. (**i**) Boxplots (see Methods for definition) showing values of PC3 to PC22 (z-scored) at laser onset of successful (REM) and unsuccessful trials (No REM). The analysis only includes trials with laser onset during NREM sleep. None of the PCs passed the significance criterion used for PC1 and PC2 in Fig. 5d; n = 20 successful, n = 25 unsuccessful trials; error bars, 95% CIs. (**j**) Mean activity of different neuron subclasses during the 50-s interval before laser onset (including only trials with laser onset during NREM). Mixed ANOVA with subclass as between and laser outcome (REM vs no REM) as within factor; interaction, P = 2.58×10^−5^, F(3, 552) = 8.14; main effect of laser outcome, P = 0.0016, F(1, 552) = 10.13; two-sided paired t-tests with Bonferroni correction, c1-c2-: P = 2.82×10^−5^, T(132) = 4.68; c1+c2+: P = 1, T(152) = -0.61; c1+c2-: P = 0.52, T(245) = 1.52; c1-c2+: P = 1, T(23) = -1.10; c1+c2+, n = 153; c1-c2-, n = 133; c1+c2-, n = 246; c1-c2+, n = 24 units. Dots, individual units. Horizontal lines, mean; error bars, 95% CIs. ***P < 0.001. Source data

Our analysis of the population activity suggests that PC2 reflects REM propensity (Fig. 2). To further test this prediction, we enhanced the likelihood of NREM→REM transitions by optogenetically activating GABAergic neurons in the dmM, while performing Neuropixels recordings in the same pontine and midbrain areas as before (Fig. 5a,b and Extended Data Fig. 7a,b). Prior research indicated an important role of the dmM in REM sleep induction through its interactions with downstream pontine and midbrain areas^10,35,50–52^. In particular, we have shown that ChR2-mediated activation of dmM GAD2 neurons strongly increases the probability of NREM→REM transitions^10^. The axons of these inhibitory neurons project to areas in the midbrain and pons, where we recorded using Neuropixels, including the PAG and dorsal PRN as well as pontine and midbrain raphe nuclei (Extended Data Fig. 7c), and have been proposed to promote REM sleep through suppression of REM-off neurons within these areas^10,16,35,50^.

**Extended Data Fig. 8:**
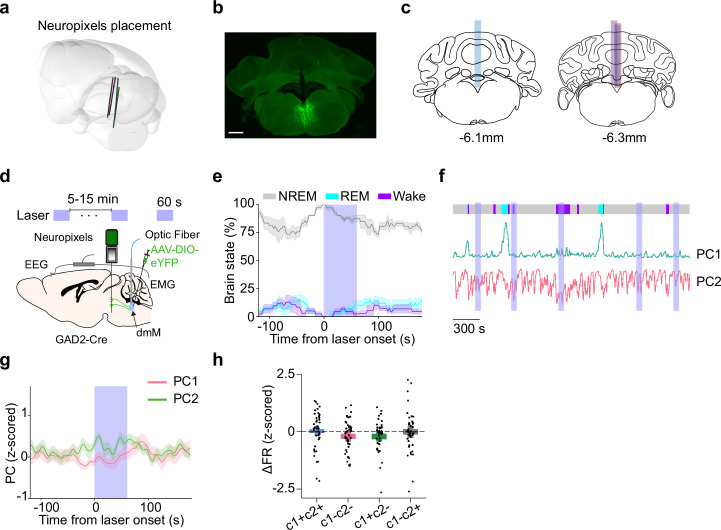
Effects of laser stimulation on midbrain/pontine population activity in eYFP mice. (**a**) Anatomical reconstruction of electrode tracts in GAD2-Cre mice injected with AAV-DIO-eYFP (n = 4). (**b**) Fluorescence images showing expression of eYFP in dmM of a GAD2-Cre mouse. (**c**) Location of optic fibers for laser stimulation. Each colored bar represents the location of an optic fiber in a GAD2-Cre mouse (n = 4 mice). (**d**) Scheme illustrating Neuropixels recordings in midbrain and pons of an eYPF mouse, while providing laser stimulation in the dmM. The laser was turned on every 5 - 15 min for 60 s (10 Hz). (**e**) Percentage of Wake, NREM, and REM sleep before, during, and after laser stimulation, including only trials with the laser onset (t = 0 s) falling on NREM sleep. Two-way RM ANOVA comparing the mean percentage of each brain state during the 60-s laser and preceding 60-s baseline interval; interaction, F(2, 6) = 0.50, P = 0.54; n = 4 mice. Shadings, s.e.m. (**f**) Example recording showing hypnogram along with PC1 and PC2. Blue shadings indicate the timing of the 60-s laser trials. (**g**) Laser-trial averaged PC1 and PC2 activity before, during, and after laser stimulation for trials where the laser onset fell on NREM (including both successful and unsuccessful trials). Two-way RM ANOVA comparing the mean value of each PC during the laser and preceding 60-s baseline interval; interaction, P = 0.3642, F(1, 40) = 0.8425; two-sided paired t-tests (Bonferroni-corrected); baseline vs laser: PC1, P = 1, T(40) = -0.14; PC2, P = 0.27, T(40) = -1.52; n = 41 laser trials. Shadings, s.e.m. (**h**) Laser-induced firing rate changes for different neuron subclasses. Two-sided one-sample test (Bonferroni-corrected) for mean firing rate changes (50 s pre- vs 5 s post-laser onset); c1-c2-: P = 0.31, T(40) = -1.81; c1+c2+: P = 1, T(40) = 0.27; c1+c2-: P = 0.61, T(40) = -1.46; c1-c2+: P = 1, W = 415; n = 41 laser trials. Horizontal lines, mean; error bars, 95% CIs. Source data

We stimulated dmM GAD2 neurons every 5 min to 15 min for 60 s at 10 Hz (Fig. 5a,b and Extended Data Fig. 7d,e), resulting in both successful trials, where the mouse transitioned from NREM to REM sleep within the laser interval, and unsuccessful trials without REM sleep (Extended Data Fig. 7f(iv)). The percentage of REM sleep was significantly increased during the laser interval compared with the 60-s baseline interval before laser onset (Extended Data Fig. 7d and Supplementary Table 1), while we observed no significant changes in control mice injected with eYFP (Extended Data Fig. 8a–e). During successful trials, REM sleep was induced with an average delay of 25.42 s ± 2.46 s. Before spontaneous and laser-induced REM sleep episodes, the population activity reached similarly high PC2 values (Extended Data Fig. 7g), suggesting that in both cases a large PC2 value in the population activity is a prerequisite to enter REM sleep, as we observed in the spontaneous activity recordings in C57BL/6J mice (Fig. 2c,e,g). The increased percentage of REM sleep during laser stimulation was reflected in an increase of PC1 and PC2 (Fig. 5c and Supplementary Table 1), whereas no significant changes were observed in eYFP control mice (Extended Data Fig. 8f,g). Consistent with the REM-promoting effect of dmM GAD2 neuron stimulation, we observed a rapid increase in the activity of REM-on (c1+c2+) neurons within 5 s of laser onset (Extended Data Fig. 7f,h), whereas the activity of neurons with negative PC2 tuning (c1−c2− and c1+c2−) decreased (Extended Data Fig. 7f,h). There were no laser-induced changes in the firing rates in eYFP mice (Extended Data Fig. 8h).

Next, to test which of the PCs best predicts the outcome of laser stimulation, we compared their activity right at laser onset. Given that optogenetic dmM GAD2 neuron stimulation promotes NREM→REM, but not Wake→REM transitions^10^, we only included trials where the mouse was in NREM sleep at laser onset. Interestingly, at the onset of successful laser trials, PC2 was on average higher than at the beginning of unsuccessful trials (Fig. 5d). In contrast, the values of PC1 as well as PC3 to PC22 at laser onset did not significantly differ between successful and unsuccessful trials (Fig. 5d and Extended Data Fig. 7i). Thus, a sufficiently large PC2 value at laser onset was required for dmM GAD2 neuron activation to induce a NREM→REM transition. In contrast, if PC2 was too low at the onset, laser stimulation likely failed to induce REM sleep (Fig. 5d). Next, to directly test whether PC2 is predictive of whether dmM GAD2 stimulation successfully initiates REM sleep or not, we predicted the outcome of dmM activation based on the values of PC1 to PC22 at laser onset using a linear classifier (Methods). Interestingly, we found that only for PC2 as input, the prediction accuracy of the classifier significantly surpassed chance level (Fig. 5e). These findings demonstrate that, among all PCs, PC2 best encodes the network’s current propensity for REM sleep. In contrast, changes in PC1 (and PC3 to PC22) only reflect post hoc changes in the brain state, as their activity at laser onset fails to predict the future outcome of dmM activation. Finally, we analyzed the activity of the different neuron subclasses before laser trials initiated during NREM sleep. Of all subclasses, only the firing rates of c1−c2− neurons significantly differed before successful compared with unsuccessful trials, with lower firing rates preceding successful laser trials (Fig. 5f and Extended Data Fig. 7j). Thus, these results suggest that the activity of REM-off neurons is a key factor in gating NREM→REM transitions in response to dmM GAD2 neuron activation.

**Extended Data Fig. 9:**
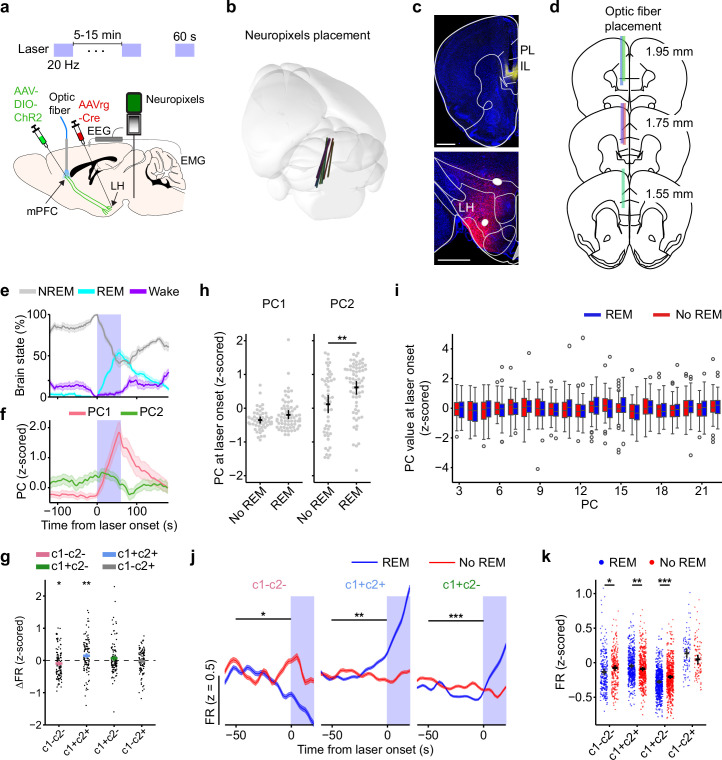
PC2 predicts REM sleep induction by mPFC→LH neuron activation. (**a**) Scheme illustrating Neuropixels recordings in midbrain and pons of a WT mouse combined with optogenetic activation of mPFC→LH neurons, targeted using retrograde AAVrg-Cre, injected into the LH, and AAV-DIO-hChR2, injected into the mPFC. The laser was turned on every 5 - 15 min for 60 s (5 Hz). (**b**) Anatomical reconstruction of Neuropixels probe tracts (8 recordings in 5 mice). (**c**) Expression of ChR2-eYFP in mPFC (top, yellow) and Cre-mCherry in LH (bottom, red). PL, prelimbic cortex; IL, infralimbic cortex; LH, lateral hypothalamus. Blue, DAPI stain. Scale bars, 1 mm. (**d**) Each colored bar represents the location of an optic fiber used for optogenetic stimulation of mPFC→LH neurons (n = 5 mice). (**e**) Percentage of Wake, NREM, and REM sleep before, during, and after optogenetic stimulation of mPFC→LH neurons, including only trials with the laser onset (t = 0 s) falling on NREM sleep. Two-way RM ANOVA comparing the mean percentage of each brain state between the laser and preceding 60-s baseline interval; interaction, P = 0.0002, F(2, 8) = 70.23; two-sided paired t-tests with Bonferroni correction; REM: P = 0.0003, T(4) = 15.88; Wake: P = 0.28, T(4) = -2.21; NREM: P = 0.0066, T(4) = -7.0; n = 5 mice. Lines, averages across mice; shadings, 95% CIs. (**f**) Laser-trial averaged PC1 and PC2 activity across trials with laser stimulation initiated during NREM sleep. Two-way RM ANOVA comparing the mean value of each PC during the laser and preceding 60-s baseline interval; interaction, P = 0.0, F(1, 118) = 43.84; two-sided paired t-tests with Bonferroni correction; baseline versus laser: PC1, P = 0.0, T(118) = 9.19; PC2, P = 0.052, T(118) = 2.26; n = 119 trials. Lines, averages across trials; shadings, 95% CIs. (**g**) Laser-induced changes in firing rates for different subclasses. Two-sided one-sample test (Bonferroni-corrected) for mean firing rate changes (50 s pre- vs 5 s post-laser onset); c1-c2-: W = 2543, P = 0.026; c1+c2+: T(118) = 3.13, P = 0.0087; c1+c2-: W = 3470, P = 1.0; c1-c2+: W = 3560, P = 1.0; n = 119 laser trials. Dots, laser trials. Horizontal lines, mean; error bars, 95% CIs. *P < 0.05, **P < 0.01. (**h**) PC1 and PC2 values at the onset of successful (REM) trials leading to REM sleep and unsuccessful (No REM) trials without REM sleep (including only trials with laser onset during NREM). Two-way ANOVA with PCs and laser outcome as between factors; interaction, P = 0.043, F(1, 234) = 4.15; two-sided t-tests with Bonferroni correction; REM versus No REM: PC1, P = 0.060, T(115.39) = -2.20; PC2, P = 0.0038, T(96.21) = -3.12; n = 68 successful, n = 51 unsuccessful trials. Horizontal lines, mean; error bars, 95% CIs. **P < 0.01. (**i**) Boxplot (Methods) showing values of PC3 to PC22 (z-scored) at laser onset of successful (REM) and unsuccessful (No REM) trials (including only trials with laser onset during NREM). None of the PCs passed the significance criterion used in (**h**); n = 68 successful, n = 51 unsuccessful trials. Error bars, 95% CIs. (**j**) Activity of c1-c2-, c1+c2+, and c1+c2- neurons relative to the onset of successful and unsuccessful laser trials (including only trials with laser onset during NREM). For detailed statistics, see (**k**). Shadings, s.e.m. *P < 0.05, **P < 0.01, ***P < 0.001. (**k**) Mean firing rates of different neuron subclasses during the 50-s interval before the onset of successful and unsuccessful laser trials (including only trials with laser onset during NREM). Mixed ANOVA with neuron subclass as between and laser outcome as within factor; interaction, P = 0.0, F(3, 1378) = 21.20; two-sided paired t-tests with Bonferroni correction; c1-c2-: P = 0.027, T(238) = 2.73; c1+c2+: P = 0.0037, F(458) = -3.33; c1+c2-: P = 0.0, T(598) = 7.68; c1-c2+: P = 0.15, T(84) = -2.11; c1-c2-, n = 239; c1+c2+, n = 459; c1+c2-, n = 599; c1-c2+, n = 85 units. Dots, units. Error bars, 95% CIs. *P < 0.05, **P < 0.01, ***P < 0.001. Source data

To test whether the capability of other REM-promoting regions to trigger REM sleep is also influenced by the ongoing PC2 activity in the midbrain and pons, we optogenetically manipulated a subpopulation of medial prefrontal cortex (mPFC) neurons, while performing Neuropixels recordings in the same midbrain/pontine areas as for dmM stimulation (Extended Data Fig. 9a–d). As previously shown, the mPFC promotes NREM→REM transitions via its projections to the lateral hypothalamus^18^ (Extended Data Fig. 9c,e), which in turn contains REM sleep-active neurons projecting to the PAG^53^. Optogenetic activation of lateral hypothalamus-projecting mPFC (mPFC→LH) neurons induced NREM→REM transitions with a delay of 28.09 s ± 1.87 s and consequently led to an increase in the percentage of REM sleep (Extended Data Fig. 9e), accompanied by corresponding changes in the PCs (Extended Data Fig. 9f). Similarly to dmM GAD2 neuron stimulation, mPFC→LH neuron excitation rapidly reduced the activity of REM-off (c1−c2−) neurons, while enhancing REM-on (c1+c2+) neuron activity within the first 5 s of laser stimulation (Extended Data Fig. 9g). As observed for dmM activation, PC2 values at laser onset were significantly higher for successful than for unsuccessful trials (Extended Data Fig. 9h), whereas the values of PC1 and PC3 to PC22 did not differ significantly between conditions (Extended Data Fig. 9h,i). As observed for dmM GAD2 neurons, the prestimulation activity of the different neuron subclasses reflected an increased infraslow component before successful trials (Extended Data Fig. 9j). Specifically, before successful trials, the activity of neurons with negative PC2 tuning (c1−c2− and c1+c2−) neurons was reduced, while REM-on (c1+c2+) neurons were more active than before failed trials (Extended Data Fig. 9j,k). In sum, our findings show that the current activity of the infraslow component predicts whether activation of either medullary or prefrontal REM-on neurons induces REM sleep.

**Extended Data Fig. 10:**
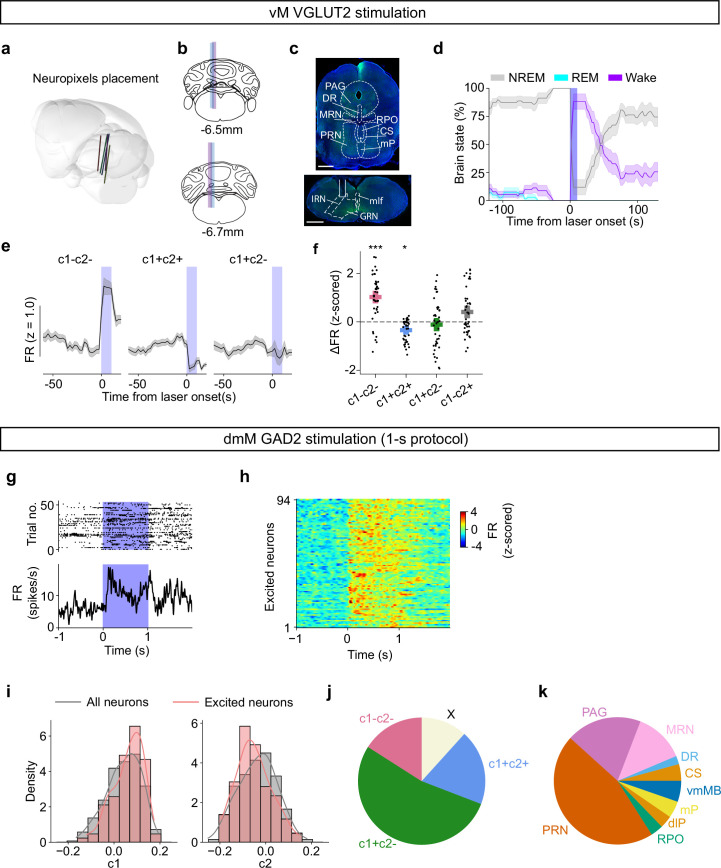
Effects of vM Vglut2 stimulation and PC1/PC2 tuning of midbrain/pontine neurons excited by dmM GAD2 neuron stimulation. (**a**) Anatomical reconstruction of electrode tracts in VGLUT2-Cre mice (5 recordings in 4 mice). (**b**) Location of optic fibers for optogenetic activation of vM VGLUT2 neurons. Each colored bar represents the location of an optic fiber in a VGLUT2-Cre mouse (n = 4 mice). (**c**) Fluorescence images showing expression of ChR2-eYFP in vM of a VGLUT2-Cre mouse (bottom) and axonal projections of vM VGLUT2 neurons in midbrain and pons (top). IRN, intermediate reticular nucleus; mlf, medial longitudinal fasciculus; GRN, gigantocellular reticular nucleus. Blue, DAPI stain. Scale bars, 1 mm. (**d**) Percentage of Wake, NREM, and REM sleep before, during, and after optogenetic stimulation of vM neurons, including only trials with the laser onset (t = 0 s) falling on NREM sleep. Two-way RM ANOVA comparing the mean percentage of each brain state during the 10-s laser and preceding 60-s baseline interval; interaction, P = 4.27×10^−4^, F(2, 8) = 114.97; two-sided paired t-tests with Bonferroni correction; REM: P = 1, T(4) = 1; Wake: P = 0.0013, T(4) = -10.79; NREM: P = 0.0013, T(4) = 10.66; n = 5 mice. Shadings, s.e.m. (**e**) Effects of vM VGLUT2 neuron excitation on the firing rates of different neuron subclasses (including only trials with laser onset during NREM). Statistical results are reported in (**f**). Solid lines, averages across laser trials (n = 42); shadings, s.e.m. (**f**) Laser-induced firing rate changes for different neuron subclasses (including only trials with laser onset during NREM). One-sample paired tests (Bonferroni-corrected) for mean firing rate changes (50 s pre- vs 5 s post-laser onset), c1-c2-: P = 6.04×10^−6^, W = 96.0; c1+c2+: P = 0.03, W = 243.0; c1-c2+: P = 0.09, W = 270.0; c1+c2-: P = 1.0, T(41) = -0.28; n = 42 laser trials. Dots, individual laser trials. Horizontal lines, mean; vertical lines, 95% CIs. *P < 0.05, ***P < 0.001. (**g**) Top, spike raster for an example unit excited by dmM GAD2 neuron activation. Bottom, average laser trial-averaged firing rate. Blue shading, 1-s laser interval. (**h**) Laser trial-averaged firing rates of excited neurons relative to laser onset. Each row in the heatmap color-codes the z-scored firing rate of a single neuron. (**i**) Histograms showing distribution of c1 and c2 coefficients of excited neurons and of the full neuron population (gray). Histograms were overlaid with KDE plots (solid lines). Two-sided Mann-Whitney U test with Bonferroni correction; c1: excited vs all units, P = 0.13, U = 27062.0; c2: P = 0.070, U = 34819.0; excited units, n = 94; all units, n = 653. (**j**) Proportion of different neuron subclasses within the population of excited neurons (n = 94). X, unclassified units. (**k**) Distribution of excited neurons (n = 94) across brain areas. Source data

Finally, to determine whether these observed changes in the population activity are specifically associated with the activation of REM-promoting neurons, we optogenetically stimulated a population of wake-promoting neurons, VGLUT2-expressing neurons in the ventral medulla (vM)^25^, while performing simultaneous Neuropixels recordings in the midbrain and pons (Fig. 5g,h and Extended Data Fig. 10a–c). Activation of vM VGLUT2 neurons reliably induced arousal, reflected by a sharp increase in wakefulness during the laser interval and suppression of both NREM and REM sleep (Extended Data Fig. 10d). At the population level, this was accompanied by a large and rapid reduction in PC2 (Fig. 5i). Consistent with this shift, stimulation of vM VGLUT2 neurons reduced the activity of REM-on (c1+c2+) neurons, while sharply increasing the activity of REM-off (c1−c2−) neurons (Extended Data Fig. 10e,f).

### dmM GAD2 neurons preferentially inhibit REM-off neurons

**Fig. 6:**
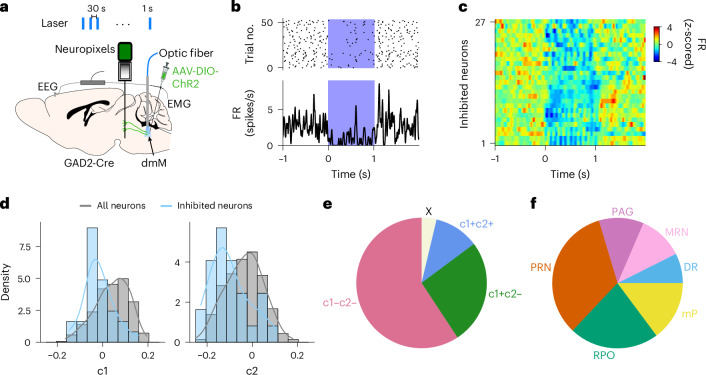
dmM GAD2 neurons preferentially inhibit REM-off neurons. **a**, Schematic illustrating Neuropixels recordings in the midbrain and pons of a GAD2-Cre mouse, while optogenetically activating dmM GAD2 neurons every 30 s for 1 s (10 Hz). **b**, Top, spike raster showing for an inhibited unit all 1-s laser trials. Bottom, average laser trial-averaged firing rate. Blue shading indicates the laser interval. **c**, Laser trial-averaged firing rates of inhibited neurons relative to laser onset (*t* = 0 s). Each row in the heat map color-codes the *z*-scored firing rate of a single unit. **d**, Histograms showing distribution of c1 and c2 coefficients of inhibited neurons and of the full neuron population (gray). Histograms were overlaid with KDE plots (solid lines). X, unclassified units. Two-sided Mann–Whitney *U*-test with Bonferroni correction; PC1: inhibited versus all units, *P* = 0.000033, *U* = 13122.50; PC2: *P* = 0.00027, *U* = 12632.50; inhibited units, *n* = 27; all units, *n* = 653. **e**, Proportion of different neuron subclasses within the population of inhibited neurons (*n* = 27). **f**, Distribution of inhibited neurons (*n* = 27) across brain areas. Source data

In principle, an increase in PC2 of the midbrain/pontine population activity can be the result of excitation of neurons with positive PC2 tuning or caused by inhibition of neurons with negative PC2 tuning. Given that dmM GAD2 neurons are inhibitory and that excitation of their projections to the recorded areas promotes REM sleep^10^, we reasoned that their activation may directly suppress the activity of neurons with negative PC2 tuning. To test this prediction, we applied a second laser protocol (at the end of the same recording sessions), where we briefly activated dmM GAD2 neurons only for 1 s every 30 s (Fig. 6a). Using this protocol, we aimed to identify neurons in the midbrain and pons that rapidly (<1 s) respond to the laser (Fig. 6b,c and Methods), before dmM GAD2 activation may induce a brain state change. Consequently, changes in neural activity were the result of direct or indirect synaptic interactions with dmM GAD2 neurons, rather than the secondary consequence of a laser-induced brain state change. Analyzing the PC1 and PC2 tuning of neurons inhibited during the 1-s laser trials, we found that the distributions of their c1 and c2 coefficients were both markedly shifted toward negative values compared with the general c1 and c2 distribution across all recorded neurons (Fig. 6d). Consistent with this, the majority of inhibited neurons belonged to the subpopulation of REM-off (c1−c2−) neurons (Fig. 6e). The inhibited neurons were distributed across multiple brain areas, with most of them located in the PRN, RPO and mP (Fig. 6f). In addition, we identified neurons excited during the 1-s laser interval (Extended Data Fig. 10g,h), most of which were located in the PRN, PAG and MRN and were part of the c1+c2− subclass (Extended Data Fig. 10j,k). Their activation is likely the result of a local disinhibitory mechanism, given the inhibitory nature of the stimulated dmM GAD2 neurons. Unlike the inhibited units, however, the excited neurons did not show any significant bias in their c1 and c2 coefficient distributions (Extended Data Fig. 10i). In sum, these findings indicate that inhibition of REM-off neurons with negative PC2 tuning by dmM GAD2 neurons causes an initial increase in the PC2 activity, setting the transition to REM sleep in motion.

## Discussion

By recording large neuronal populations across the midbrain and pons, we identified shared activity components spanning these regions (Fig. 1). Despite substantial single-neuron heterogeneity, these components explained a large fraction of population variance, with PC2 specifically capturing REM sleep propensity (Fig. 2). Relating these population-level components back to single-neuron activity revealed subpopulations with antagonistic tuning across infraslow and ultradian timescales, reinforced by opposing functional interactions (Figs. 3 and 4). Causal manipulations showed that activating dmM REM-on neurons preferentially inhibited REM-off neurons and increased PC2, whereas activating wake-promoting vM neurons rapidly reduced PC2 by recruiting REM-off neurons (Figs. 5 and 6). Importantly, the momentary strength of PC2 further influenced how upstream inputs from REM-on neurons in the medulla and mPFC affected REM sleep initiation (Fig. 5 and Extended Data Fig. 9). Together, these findings indicate that whether and when REM sleep is initiated depends not only on the activation or suppression of specific REM-on or REM-off populations, but also critically on the current midbrain–pontine network state captured by PC2.

Current mathematical models of REM sleep control propose that transitions into and out of REM sleep are governed by a bistable network, in which mutually inhibitory REM-on and REM-off neurons stabilize the network state in either NREM or REM sleep^54–58^. This framework originated from the discovery of mutual inhibition between REM-off neurons in the vlPAG/DpMe and REM sleep-active neurons in the dlP^24^. Our findings suggest that such bistable dynamics are not restricted to this specific circuit but instead emerge from distributed local interactions across midbrain and pontine regions, with particularly dense interconnections in the pons. These antagonistic interactions pit competing REM-promoting and REM-suppressing populations against each other along the PC2 dimension. In the bistable model, NREM→REM switches are thought to result from slow ultradian increases in REM-on activity or decreases in REM-off activity, reflecting accumulating homeostatic REM sleep pressure^2,15,54,55^. In line with this, we observed opposing ramping of REM-on and REM-off populations during NREM sleep across the inter-REM interval, thereby progressively biasing the network toward REM sleep. Although ultradian modulations have previously been described in vlPAG GABAergic and DR serotonergic neurons^12,15^, our results suggest that this ramping is a broader feature of REM-off and REM-on populations across midbrain and pons and is tightly coupled to infraslow fluctuations (Fig. 4f). As time since the last REM episode elapses, the REM-off infraslow modulation wanes, whereas the REM-on baseline activity rises (Fig. 4g), reflecting accumulating REM pressure and increasing the likelihood of NREM→REM transitions. Thus, the current state of the network, influenced by intertwined infraslow and ultradian processes, sets the overall propensity to enter REM sleep.

Because they are minimally active during REM sleep and negatively correlated with EEG *σ* power^9,11–13,59,60^, both locus coeruleus noradrenergic and DR serotonergic neurons fall into the c1−c2− (REM-off) subclass. Fluctuating norepinephrine and serotonin levels likely entrain downstream receptor-expressing neurons, contributing to the widespread infraslow rhythmicity observed across brainstem populations and in other regions including the preoptic area, hippocampus and cortex^14,61–63^. The strong self-excitation among neurons with negative PC2 tuning (Fig. 3f) likely further reinforces this rhythm. Local inhibition of REM-on by REM-off neurons (Fig. 3d) may contribute to the strong suppression of REM sleep caused by activating neurons falling into the c1−c2− subclass including locus coeruleus noradrenergic, DR serotonergic and vlPAG/DpMe GABAergic neurons^9,11,15,32,33^. Previous studies showed that norepinephrine regulates spindle frequency and *σ* power^9,59^, indicating that reduced locus coeruleus activity and declining norepinephrine levels before REM sleep contribute to the rise in *σ* power before NREM→REM transitions^9,11,13,59,64^. Our study further provides correlational evidence that neurons with negative infraslow rhythmicity lead cortical *σ* power (Fig. 3b). This temporal relationship may help explain why the *σ* power predicts imminent transitions to REM sleep^4,64,65^. Because acetylcholine levels fluctuate inversely with *σ* power^60,66^ and pontine cholinergic neurons are activated during both REM sleep and wakefulness^47–49^, these neurons are likely part of the c1+c2− subclass (Extended Data Fig. 5b).

The antagonistic infraslow dynamics of REM-on (c1+c2+) and REM-off (c1−c2−) neurons create minute-scale windows in which NREM→REM transitions can occur^9^. Transitions are most likely at the ISO peak, when REM-on activity is maximal and REM-off neuron firing is reduced. In support of this, optogenetic locus coeruleus inhibition most effectively promoted REM sleep entries when the *σ* power was descending toward its trough (where locus coeruleus noradrenergic activity is highest), whereas activation of these REM-off neurons at the rising ISO phase blocked transitions to REM sleep^9^. However, not all ISO cycles result in a spontaneous NREM→REM transition. Whether a given cycle leads to REM sleep likely depends on the momentary balance between REM-on and REM-off populations, shaped by ultradian ramping dynamics and external inputs. When the REM transition fails, the animal may instead transition to wakefulness, consistent with the increase in PC2 preceding NREM→Wake transitions, although less strongly than before NREM → REM transitions (Extended Data Fig. 3c).

Any upstream circuit enhancing PC2, by either inhibiting REM-off or exciting REM-on neurons, should tip the balance toward REM sleep. Conversely, populations that suppress PC2 are expected to inhibit REM sleep, as observed for vM VGLUT2 neurons (Fig. 5g–i). Their strong activation of REM-off neurons likely contributes to suppressing Wake→REM transitions. Prevalent models propose that REM sleep is induced by disinhibition, whereby upstream REM-on neurons inhibit the midbrain REM-off neurons and thereby disinhibit brainstem neurons generating the defining REM sleep features^1,3^. Alternatively, a recent study showed that excitatory projections from the medulla to the dlP also promote REM sleep by exciting REM-on neurons in the sublaterodorsal tegmentum^52^. Our study provides in vivo evidence for a disinhibitory mechanism via dmM GAD2 neuron activation. First, REM-off neurons in the midbrain and pons led REM-on neurons in time (Extended Data Fig. 5d), and functional connectivity analysis suggests that REM-promoting neurons with positive PC2 tuning are under strong inhibitory control by negatively tuned neurons (Fig. 3d). Strong self-excitation within the REM-off population may further exacerbate their dominance, whereas self-inhibition may stabilize the network. Second, dmM GAD2 neurons preferentially inhibited REM-off (c1−c2−) neurons (Fig. 6e), likely explaining why the prestimulation activity of REM-off neurons predicted whether laser stimulation triggered REM sleep (Fig. 5f). With mPFC stimulation, both REM-on activity and activity of neurons with negative PC2 tuning predicted the optogenetic outcome (Extended Data Fig. 9j,k), suggesting that mPFC→LH neurons, or their downstream hypothalamic targets^18^, act on both REM-on and REM-off neurons in the midbrain and pons. Nevertheless, both dmM and mPFC→LH neuron stimulation produced the same effect: rapid suppression of REM-off neurons together with activation of REM-on neurons (Extended Data Figs. 7h and 9g), consistent with local connectivity that reinforces their antagonistic balance.

Disentangling neural populations that are directly inhibited by the dmM, or recruited via the mPFC→LH pathway, and that causally mediate NREM → REM transitions, from those that passively follow the network dynamics due to their connectivity will be important for future research. Our analysis suggests that the key neurons driving the observed dynamics are negatively correlated with PC2 (consistent with REM-off neurons leading REM-on neuron activity), show strong ultradian ramping activity that mirrors REM pressure accumulation, and, based on mathematical models of the NREM→REM switch^54,55^, are expected to reciprocally inhibit REM-on neurons. Given the particularly dense interconnections between neurons with opposing PC2 tuning in the pons (Extended Data Fig. 4h), such neurons are likely concentrated there. Consistent with this, we found most neurons inhibited by dmM GAD2 neurons in pontine areas (Fig. 6f).

A limitation of our study is that recordings were performed in head-fixed animals. Determining how key population features, such as infraslow rhythmicity and ultradian ramping, are shaped by waking experiences or allostatic challenges such as stress will require recordings in freely moving animals. Another important question is whether the dynamics observed here are brainstem specific or shared by REM sleep-regulatory populations across subcortical and cortical regions, and how such dynamics are coordinated across the brain. Our study provides a conceptual framework for comparing the low-dimensional dynamics that gate REM sleep across different regions. Because few studies have linked population dynamics to single-neuron properties and their connectivity^67–69^, future work should disentangle the anatomical and synaptic network structure from which the observed dynamics emerge. Given their low-dimensional structure and interpretability, brainstem sleep dynamics are well suited to causally relate network architecture with dynamic principles governing the NREM–REM cycle.

## Methods

### Animals

All experiments were approved by the Institutional Animal Care and Use Committee at the University of Pennsylvania and conducted in accordance with the guidelines set by the National Institutes of Health (NIH) Office of Laboratory Animal Welfare Policy. Experiments were performed in GAD2-IRES-Cre mice (Jackson Laboratory stock no. 010802), C57BL/6 mice (Jackson Laboratory stock no. 000664) and VGLUT2-IRES-Cre mice (Jackson Laboratory stock no. 016963). The number of animals per cohort and the sex of each animal are reported in Supplementary Table 2; most animals were male. Animals were housed on a 12-h light–dark cycle (lights on at 7:00 and off at 19:00) and were aged 8–12 weeks at the time of surgery. The colony room was maintained at an ambient temperature of 20–23 °C and a humidity of 40–60%. All mice were group housed with free access to food and water.

### Surgical procedures

All surgeries were performed following the Institutional Animal Care and Use Committee guidelines for rodent survival surgery. Before surgery, mice were given meloxicam subcutaneously (5 mg per kg body weight). Mice were anesthetized using isoflurane (1–4%) and placed in a stereotaxic frame with a heating pad to maintain the body temperature during the procedure. After asepsis, the skin was incised to expose the skull. The skull location on top of the midbrain/pontine recording location was marked with a permanent marker (−4.6 mm AP, 0–0.6 mm ML). A headplate was then affixed over the skull with dental cement, and the exposed skull was protected with Quik-Cast. To manipulate dmM GAD2 neurons, GAD2-IRES-Cre mice were injected before the headplate implantation with AAV1-EF1α-DIO-hChR2-eYFP-WPRE-hGH (0.1–0.3 μl, University of Pennsylvania Vector Core, RRID: Addgene_20298) into the dmM using Nanoject II (Drummond Scientific) via a glass micropipette (−3.6 mm DV, 0 mm ML, −6.4 mm AP). Control eYFP animals were instead injected with AAV2-EF1-α-DIO-eYFP-WPRE-hGH (0.1–0.3 μl, UNC Vector Core, RRID: Addgene_27056) into the dmM. For vM VGLUT2 stimulation, VGLUT2-IRES-Cre mice were injected with AAV5-EF1α-DIO-hChR2-eYFP-WPRE-hGH (0.1–0.2 μl University of Pennsylvania Vector Core, RRID: Addgene_20298) into the vM (−4.7 mm DV, −0.7 mm ML, −6.4 mm AP). To optogenetically manipulate mPFC→LH neurons, we injected AAVrg-Ef1a-mCherry-IRES-Cre (0.35 μl, RRID: Addgene_55632) into the lateral hypothalamus (−1.4 mm AP, 1 mm ML, −5.2 mm DV), followed by AAV2-EF1a-DIO-hChR2(H124R)-eYFP (0.4 μl, UNC vector core, lot no. AV4378Q) injection into the mPFC (1.85 mm AP, 0.35 mm ML, 2.3 mm DV). After virus injection, an optic fiber (0.2 mm in diameter) was inserted into the dmM (DV −3.4 mm to −3.5 mm), vM (4.65 mm DV) or mPFC (2.3 mm DV) for optogenetic stimulation. EEG signals were recorded using stainless-steel wires attached to two screws, one on top of the parietal cortex and one on top of the frontal cortex. The reference screw was inserted on top of the left cerebellum. For EMG recordings, two stainless-steel wires were inserted into the neck muscles. All electrodes, screws and connectors were secured to the skull using dental cement. After surgery, bupivacaine (2 mg per kg body weight) was administered at the incision site, and we monitored any signs of pain or distress every 10 min until animals were fully recovered from anesthesia. Afterward, mice were further monitored at least once daily for 3 days and then at least twice weekly for any signs of pain or distress and changes in body weight.

### Habituation to head fixation and polysomnographic recordings

One to two weeks after surgery, mice were habituated to sleep well under head fixation. Habituation began with 30–60 min of head fixation on the first day, and the duration was subsequently increased each day until up to 5 h during the last days. The full habituation period lasted 14–21 days^18^. Before the actual recording day, mice underwent a 4-h sleep session in the head-fixed setup (without Neuropixels probe insertion) to confirm that they can sleep well in head fixation. All sleep sessions were conducted during the light phase (8:00–18:00). EEG and EMG signals were recorded using an RHD2132 amplifier (Intan Technologies, sampling rate 30 kHz) connected to an RHD USB Interface Board (Intan Technologies), which was controlled using the OpenEphys software (Rhythm FGPA plugin). Signals were referenced to a common ground screw, inserted on top of the cerebellum. During the recordings, EEG and EMG electrodes were connected to flexible recording cables using a small connector.

For further processing, EEG and EMG signals were downsampled to 1 kHz. Brain states were scored manually by visual inspection of the EEG and EMG signals, EEG spectrograms and EMG power using a graphical user interface programmed in Python (https://github.com/tortugar/Lab/tree/master/PySleep/). EEG and EMG spectrograms were computed using consecutive fast Fourier transforms with a Hanning window, calculated for sliding, half-overlapping 5-s windows resulting in a 2.5-s time resolution of the hypnogram. The EMG power was computed by integrating the EMG spectrogram in the range of 10–500 Hz. States with low amplitude, fast EEG activity and increased EMG tone were scored as Wake. States with dominant theta (*θ*) oscillations, low delta (*δ*) power and minimal EMG tone were scored as REM sleep. States with high-amplitude *δ* activity and low EMG tone were scored as NREM sleep.

### Electrophysiological recordings

The day before the Neuropixels recordings, mice were anesthetized, and a small (<1 mm) craniotomy was made over the premarked location, and the exposed skull was covered with Quik-Cast. Mice were then allowed to recover in their home cage until the time of recording. The next day, mice were head-fixed to the recording apparatus. The Quik-Cast was removed, and a pool of saline was put into the craniotomy site within the headplate to keep the craniotomy moist. Recordings were performed using Neuropixels 1.0 probes with the reference and ground shorted together. Before the recording, the Neuropixels probe was coated with red or green fixable lipophilic dye (CM-DiI, Thermo Fisher) and allowed to dry. The Neuropixels probe reference was connected to the same implanted reference screw as that used for the EEG/EMG electrodes. A flexible recording cable was connected to the EEG and EMG electrodes. The Neuropixels probe was positioned over the craniotomy using a micromanipulator (Sensapex) and was slowly (2 µm s^−1^) inserted into the brain (4.5–5 mm), while being observed under a stereomicroscope to prevent bending or bleeding. Once the target depth was reached the probe was retracted 100 µm and the probe was allowed to sit until the recording began. Recordings started when the first REM sleep bout was observed. To record the neural data, we used OpenEphys with the Neuropixels PXI plugin, running on an acquisition computer connected to the PXI chassis (PXIe-1071) containing the Neuropixels base station. The EEG/EMG data were recorded simultaneously using the Intan amplifier via the Rhythm FPGA OpenEphys plugin. A sync pulse was sent from the Neuropixels base station and recorded on the digital input of the Intan board, as well as a digital input of a National Instrument BNC board (National Instruments, BNC 2110) connected to an I/O module (PXI-6133) in the PXI chassis. This was used to synchronize the timing between the Neuropixels and Intan signals. For optogenetic stimulation sessions, a blue 473-nm laser (Laserglow; 4–6 mW; 2 mW for mPFC→LH neuron stimulation) was used to stimulate for 60 s at 10 Hz (5 Hz for mPFC→LH neurons) with 10-ms pulse width in a raised cosine pattern with the interstimulation interval randomly chosen between 5 min and 15 min. The 60-s stimulation protocol lasted for ~2.5 h. In dmM ChR2 and eYFP mice, the 60-s protocol was followed for the last 30 min of the recording by 1-s pulse trains presented every 30 s. In vM mice, we stimulated vM VGLUT2 neurons every 10–15 min for 10 s. We chose longer interstimulation intervals and shorter laser stimulation periods because of the strong wake-promoting effect of vM VGLUT2 neuron activation. In some animals, we re-covered the craniotomy with Quick-Cast and performed a second Neuropixels recording on another day. For both sessions, the probe was labeled with differently colored DiI to distinguish between recordings.

### Histology

Mice were deeply anesthetized and transcardially perfused with 0.1 M phosphate-buffered saline (PBS) followed by 4% paraformaldehyde in PBS. After removal, brains remained overnight in fixative and were then stored in 30% sucrose by volume in PBS solution for at least one night. After embedding and freezing, brains were sliced into 40-μm sections using a cryostat and mounted onto glass slides. For immunohistochemistry, brain sections were washed in PBS, permeabilized using PBST (0.3% Triton X-100 in PBS) for 30 min and then incubated in blocking solution (5% normal donkey serum in PBST, Jackson ImmunoResearch Laboratories, 017-000-001) for 1 h. To stain eYFP-expressing axon fibers, brain sections were subsequently incubated with a chicken anti-GFP primary antibody (Aves Lab, GFP8794984; 1:1,000 dilution) diluted in the blocking solution for one night at 4 °C. The next day, brain sections were washed in PBS and incubated for 2 h with a species-specific secondary antibody conjugated with red or green Alexa fluorophore (Jackson ImmunoResearch Laboratories, 703-585-155 or 703-545-155; 1:500 dilution; donkey anti-chicken) diluted in PBS. The slices were washed with PBS followed by counterstaining with Hoechst solution (Thermo Scientific) and cover-slipped with Fluoromount-G (Southern Biotechnic). Fluorescence images were taken using a fluorescence microscope.

### Electrode tract reconstruction

To reconstruct the Neuropixels probe tract, we first registered each brain section with a DiI probe track to the Allen Common Coordinate Framework using the software SHARP-track (https://github.com/cortex-lab/allenCCF/)^38^. Next, we manually outlined the DiI-labeled segment of the Neuropixels probe tract on each registered brain section. Lastly, a 3D reconstruction of the probe trajectory was fitted to the traced probe segments using SHARP-track. Neuropixels channels were then manually aligned to anatomical features along the probe trajectory using electrophysiological landmarks using the International Brain Lab electrophysiology alignment tool (https://github.com/int-brain-lab/iblapps/tree/master/atlaselectrophysiology/)^70^. Visualization of probe trajectories and location of the recorded neurons in the 3D reference mouse brain was performed using Urchin (https://github.com/VirtualBrainLab/Urchin/). All analyzed units were located in the following regions: PAG, DR, MRN, RPO, CS and PRN (including both PRNc and PRNr). The area ventral to the DR or PAG (labeled as midbrain (MB) in Allen atlas) was referred to as the vmMB, and the area ventral to the CS or RPO (pons (P) in Allen atlas) was denoted as the mP. Finally, the pontine area between the MRN and PRN (P in Allen atlas) was labeled as the dlP. Units in areas dorsal to the PAG (such as superior colliculus) or in areas from which we recorded only in a single mouse were excluded from the dataset.

### Spike sorting

Spike sorting was performed using Kilosort2 (ref. ^71^) or Kilosort4 (ref. ^72^). Clusters identified by Kilosort were manually curated in Phy (https://github.com/cortex-lab/phy/). Units containing low-amplitude spikes, abnormal or inconsistent waveform shapes, or refractory period violations were labeled as noise or multiunit activity and excluded from further analysis. Units were merged with similar nearby units, if the refractory period, as verified by their cross-correlogram, and waveform shape were maintained after the merge. Only units that displayed proper spike amplitude, stable waveform and refractory period were classified as ‘good’ single-neuron units and used for analysis. For each unit, the spike time points were encoded as spike trains binned in 1 ms. To calculate firing rates, spike trains were downsampled to 2.5-s bins (by computing the number of spikes per second within each bin), smoothed using a Gaussian kernel with a standard deviation of 1.5 time bins and detrended by removing linear trends across the entire recording to correct for potential drifts. Finally, we screened the firing rates of each unit across the entire recording. Units with a strong drift (despite detrending) or that suddenly stopped firing for long time intervals were excluded.

### Identification of laser-modulated units

Using the 1-s stimulation protocol, we identified units whose activity was significantly changed by dmM GAD2 neuron stimulation. We represented the activity of each unit as a 1-ms spike train. Using the Wilcoxon signed-rank test (*α* = 0.01), we then compared for each unit the average firing rate during the 1-s interval preceding laser onset with that during the 1-s laser interval. To ensure that the detected waveforms of excited units are not the result of light artifacts, we additionally required for excited units that the correlation (measured using Pearson correlation) between spike waveforms occurring within 20 ms of the onset of each laser pulse and spontaneous spikes occurring within 1-s intervals preceding each laser trial was larger than 0.95. To assess the false positive rate of this statistical approach, we applied the same analysis to eYFP control mice using identical statistical criteria. Of 564 recorded units in 4 eYFP mice, only 7 were classified as laser modulated, corresponding to a false positive rate of ~1.24%. In previous studies^10,18,25^, we showed in eYFP control experiments that laser delivery to the mPFC, dmM or vM does not alter the brain state, as determined by EEG/EMG recordings. In the present study, to assess potential effects of laser stimulation on single-unit activity, we performed Neuropixels recordings in GAD2-Cre mice expressing eYFP in the dmM and observed no laser-induced changes (Extended Data Fig. 8). We did not perform additional eYFP control experiments for vM VGLUT2 or mPFC→LH neuron stimulation combined with Neuropixels recordings in the midbrain/pons. In these experiments, the stimulated upstream regions (vM and mPFC) were located at distances from the pontine/midbrain recording site that were comparable to or greater than those for dmM stimulation, making direct laser-induced effects on recorded single-unit activity unlikely.

### Dimensionality reduction using PCA

We used singular value decomposition (SVD) to calculate for each animal the PCs across the entire recording session. Firing rates were discretized in 2.5-s bins, *z*-scored and smoothed using a Gaussian kernel with a standard deviation of 1.5 time bins. For PCA, we built a matrix ${\bf{R}}$ with the rows corresponding to all neurons (variables) and columns corresponding to the number of 2.5-s time bins within a recording session (samples). To focus the PCs on sleep activity, we excluded long wake blocks, defined as wake episodes longer than 1 min, potentially separated by sleep intervals < 20 s. Using SVD, we decomposed the matrix into ${\mathbf{R}}={\mathbf{U}}\; {\mathbf{S}}\;{{\mathbf{V}}}^{{\boldsymbol{T}}}$. The PCs correspond to the rows of the matrix ${\bf{S}}\,{{\bf{V}}}^{T}$. The *i*-th row of ${\bf{U}}$ contains the coefficients ${c1}_{i},{c2}_{i},\ldots$, which determine how strongly each PC contributes to the activity of neuron *i*. The coefficients *ck*_*i*_ are subject to the constraint ${\sum }_{i}{({{ck}}_{i})}^{2}\,=\,1$. Thus, if a given PC was correlated only with a small group of neurons, their coefficient would approach ±1. In contrast, if a PC is widely shared across the neural population, the absolute value of the coefficients is clearly smaller than 1. The PCs are unique except for their sign. For consistency across mice, we therefore set the sign of PC1 such that the average activity of PC1 was positive during REM sleep and for PC2 such that it was positively aligned with the EEG *σ* power during NREM sleep. The code for PCA is available under https://github.com/tortugar/Npx/.

### Definition of neuron subclasses

Approximating the firing rates using the first two PCs allows representing the firing rates of each unit *i* in terms of the two coefficients c1 and c2, that is, ${{\bf{fr}}}_{i}\,=\,{c1}_{i}\times {\rm{PC}}1+\,{{\rm{c}}2}_{{\rm{i}}}\times {\rm{PC}}2$. We subdivided the units whose firing rates were significantly correlated with both PC1 and PC2 (*P* < 0.05, Pearson correlation) based on the sign of their c1 and c2 coefficients into four different subclasses. As units with negative c1, but positive c2 (c1−c2+ neurons) were scarce, we focused our further analyses on neurons in the remaining three quadrants (c1−c2−, c1−c2+ and c1+c2+ neurons). To test when the *z*-scored activity in a subclass significantly deviated from baseline before NREM→REM transitions, we downsampled the firing rates to 5-s bins. We then compared the activity of each unit during the baseline interval from −120 s to −115 s to each subsequent time bin up to 0 s using paired *t*-tests with Bonferroni correction. Finally, we identified the time point (*t*_sig_) from when the *P* values were consistently <0.05 until the transition at *t* = 0 s.

### CVEV

To estimate the variance explained by the first *k* PCs, we used a bi-cross-validated version of PCA^40–42^. First, we randomly selected 70% of the time bins as training data ${{\bf{X}}}^{{tr}}$ and the remaining 30% as test set (${{\bf{X}}}^{{te}}$). Using SVD, we represented ${{\bf{X}}}^{{tr}}$ as equation (1):

1 $${{\bf{X}}}^{{tr}}={{\bf{U}}}^{k}{{\bf{S}}}^{{tr}}{{\bf{V}}}^{T,{tr}}$$

where we retained only the first *k* PCs (rows in matrix ${\bf{S}}{{\bf{V}}}^{T}$) and the corresponding coefficients (${{\bf{U}}}^{k}$, first *k* columns of **U**). For cross-validation, we split the test set along the rows (neuron dimension) into another training (80% of neurons, ${{\bf{X}}}_{1}^{{te}}$) and test set (remaining 20% of neurons, ${{\bf{X}}}_{2}^{{te}}$). The low-rank approximation (using *k* PCs) can be rewritten in block format as equation (2):

2 $$\left(\begin{array}{l}{{\bf{X}}}_{1}^{te}\\ {{\bf{X}}}_{2}^{te}\end{array}\right)\begin{array}{l}=\left(\begin{array}{l}{{\bf{U}}}_{1}^{k}\\ {{\bf{U}}}_{2}^{k}\end{array}\right){{\bf{S}}}^{{te}}{{\bf{V}}}^{T,{te}}\end{array}$$

Using ${{\bf{U}}}_{1}^{k}$ (calculated using the training data), we estimate the latent PCs in the test data, and using this estimate we then predict the firing rates in ${{\bf{X}}}_{2}^{{te}}$. The approximation for ${{\bf{X}}}_{2}^{{te}}$ is given by equation (3):

3 $${\hat{{\bf{X}}}}_{2}^{{te}}={{\bf{U}}}_{2}^{k}{\rm{pinv}}({{\bf{U}}}_{1}^{k})\,{{\bf{X}}}_{1}^{{te}}$$

where ${\rm{pinv}}()$ denotes the pseudo-inverse of a matrix. Finally, we determined how much of the variance in ${{\bf{X}}}_{2}^{{te}}$ was explained by the approximation with *k* PCs. The average explained variance by the reconstruction of the test data using the first *k* PCs was calculated over 50 repeats of the procedure, each time with randomly selected training and test sets. As the data in ${{\bf{X}}}_{2}^{{te}}$ are explained using a different set of neurons (contained within ${{\bf{X}}}_{1}^{{te}}$), the estimated PCs can in principle only explain variability shared across at least two neurons. As shown in previous studies, increasing the number of PCs (*k*) initially increases the explained variance, but it then starts dropping due to overfitting to noise or variability that is not shared across neurons, but instead unique to individual neurons (private variability)^40,41^. Consequently, the peak in the relationship between *k* and the variance explained constitutes a lower bound on the dimensionality of the shared population variability. Of note, the dimensionality of the population activity depends on the number of simultaneously recorded neurons (Supplementary Fig. 3a,b). However, by sampling fixed-sized subsamples of neurons, we found that the fraction of the shared variance explained by the first two PCs was largely independent of the number of neurons (Supplementary Fig. 3c).

### Definition of subspaces

To define within the 2D state space the regions (subspaces) of NREM, REM and Wake, we fitted a 2D Gaussian to all points within the state space labeled as NREM, REM or Wake (based on EEG/EMG annotation). The axes of the ellipses capturing the distribution of each state were determined using the covariance matrices for each state. To capture 90% of the distribution, we scaled each eigenvector by the factor ${c}_{i}=2\times 1.645\times \sqrt{{s}_{i}}$, where ${s}_{i}$ denotes the *i*-th eigenvalue of the covariance matrix. The two scaled eigenvectors correspond to the two axes of the ellipse. The eigenvector with the larger eigenvalue corresponds to the direction of the principal axis of the ellipse, which we used to determine the angles at which the neural trajectories left or returned to the NREM subspace (Fig. 2e).

### LDA

To test whether the population activity is predictive of whether the animal will transition from NREM to REM sleep or to wake, we performed a classification analysis (Fig. 2h). For each NREM→REM or NREM→Wake transition, we extracted the preceding 150 s of PC1 to PC22. Using LDA, we then tested for each PC separately how long before the transition the type of transition (NREM→REM or NREM→Wake) can be predicted using the PC activity in each 10-s bin from −150 s to 0 s. To evaluate the accuracy of the prediction, we performed Monte Carlo cross-validation. We built a training set by randomly selecting 70% of the NREM→REM trials. As the trials with NREM→Wake transitions outnumbered NREM→REM trials, we randomly selected the same number of NREM→Wake trials to balance the dataset. The remaining 30% of NREM→REM trials and the same number of randomly selected NREM→Wake trials served as the test set. Using a given training set, we then fitted the model for each preceding 10-s time bin and evaluated its performance on the test set. This procedure was repeated 25 times. We evaluated for each time point whether the LDA performance was above chance level using a one-sided *t*-test with Bonferroni correction (*P* < 0.05). Finally, we determined the time point *t*_sig_ from which the prediction accuracy consistently differed from chance level until *t* = 0 s.

To test whether the population activity at laser onset is predictive of whether optogenetic activation will induce REM sleep or not, we again performed LDA (Fig. 5e). We first divided the laser trials into two categories: successful trials, where REM sleep occurred within the following 60-s interval, and unsuccessful trials without REM sleep. To build the dataset for training and testing the model, we extracted the values of each PC from PC1 to PC22 at laser onset across all mice. We then randomly selected 70% of the trials as the training set to fit the model and used the remaining 30% as the test set to evaluate its performance. This procedure was repeated ten times for each PC.

### Neuron activity during infraslow cycles and inter-REM

To average the firing rates of single units across multiple ISO cycles, we determined from the EEG *σ* power the beginning and end of each ISO cycle within consolidated bouts of NREM sleep (≥90 s duration including microarousals, defined as wake bouts < 20 s). For this, we first calculated the EEG power spectrogram using fast Fourier transforms in sliding, half-overlapping 5-s windows. Next, we smoothed the EEG spectrogram along the time dimension using a 12.5-s box filter, normalized each frequency component by its mean, calculated the power in the *σ* range, and then band-pass filtered the *σ* power in the range of 1/80–1/30 Hz using a 4th-order digital Butterworth filter. Finally, we computed the phase angle by applying the Hilbert transform to the band-pass filtered *σ* power signal. Based on the phase, we could then isolate the beginning and end of single cycles, which allowed us to normalize the duration of single ISO cycles in time and to average the activity of single units across multiple ISO cycles. To measure the strength (amplitude) of the infraslow modulation of a given unit, we calculated the difference between its maximum and minimum firing rates within ISO cycles.

To analyze the activity of units throughout the inter-REM interval (interval from the end of a REM sleep episode until the beginning of the next one), we normalized the duration of all inter-REM intervals allowing us to average the activity of neurons across multiple intervals. For time normalization, we resampled the firing rate vector (2.5-s binning) within each inter-REM interval to ten bins. To determine the slope at which the activity of each neuron increased or decayed throughout inter-REM, we performed linear regression between the time bins along the normalized time axis and the mean *z*-scored firing rates for each bin. To avoid outliers at the beginning or end of inter-REM, we excluded the first and last bin of the inter-REM interval for the regression fit.

To analyze specifically how the wake or NREM activity of neurons evolved across inter-REM, we resampled both the firing rates and hypnogram vectors within each inter-REM interval to ten bins. For each bin, we determined the average NREM or wake activity. As before, we used linear regression (after excluding the first and last bin of the inter-REM intervals) to calculate the slope at which the activity changed throughout inter-REM. The analyses in Fig. 4 include all 43 inter-REM intervals from six mice (Extended Data Fig. 6b).

To analyze how the infraslow fluctuations in neural firing rates changed throughout inter-REM, we divided inter-REM intervals into three (equally sized) sections. We then quantified, for each unit, how both the amplitude of its infraslow modulation (difference between the maximum and minimum average firing rates during ISO cycles) and its minimum activity during ISO cycles changed across the three sections. The beginning and start of ISO cycles was determined from the EEG *σ* power. We required that the inter-REM duration was at least 270-s long (corresponding to three sections with at least 90 s in duration), leading to the exclusion of one inter-REM interval. We used ANOVA followed by pairwise *t*-tests to assess whether the amplitude or minimum activity showed a significant progressive trend across inter-REM sections, defined as either a monotonic increase (early < middle < late) or a monotonic decrease (early > middle > late).

### Cross-correlation between single units, *σ* power and PC2

To calculate the cross-correlation between different neuron subclasses during NREM sleep, we discretized their firing rates in 250-ms bins and smoothed them using a Gaussian kernel with a standard deviation of 1.5 time bins. For each pair of units, we calculated the cross-correlation of their firing rates for each NREM episode with duration ≥ 120 s (including microarousals), and then averaged across NREM sleep episodes. The cross-correlation was normalized by dividing it by the product of the standard deviations of two correlated signals times their length. To cross-correlate PC2 and EEG *σ* power, we calculated the normalized cross-correlation of PC2 with the *σ* power for each NREM episode with duration ≥ 120 s. Both signals were binned in 2.5 s. Finally, we averaged the cross-correlograms across all NREM episodes.

To calculate the cross-correlation between the firing rates of individual neurons and *σ* power, we first calculated for all NREM episodes with duration ≥ 120 s the *σ* power from the EEG spectrogram, computed using consecutive 2.5-s windows with 90% overlap, yielding a 250-ms temporal resolution. We again normalized the spectrogram by dividing each frequency component by its mean power. Using the same overlapping binning, we downsampled the 1-ms spike trains to firing rates to prevent any time lags resulting from differences in downsampling and then calculated the cross-correlation of both signals. The cross-correlation was normalized as described above. For each unit, we finally obtained the mean cross-correlation by averaging across all NREM episodes.

### Functional connectivity analysis

To evaluate functional connectivity between simultaneously recorded neurons on the synaptic timescale, we computed jitter-corrected CCGs following previous studies^44–46^. Spike trains of each pair of neurons from the same recording were discretized at 1-ms resolution and cross-correlated over the entire recording to obtain the raw CCG (Extended Data Fig. 4a,b). Next, we constructed a jittered CCG by shuffling the time points of the spikes within consecutive 30-ms windows (jitter window) to disrupt correlations on a timescale of ≤30 ms, while maintaining slower correlations. The jitter-corrected CCG was finally obtained by subtracting the jittered CCG from the raw CCG and normalized by the geometric mean of the total spike counts of the two neurons. By subtracting the jittered cross-correlogram from the original one, we removed correlations on slower (>30 ms) timescales, which may reflect shared inputs or global fluctuations in activity. To quantify the strength of potential interactions, we calculated the maximum positive or negative peak within the ±20-ms window centered around zero. The peaks were normalized by the standard deviation of the CCG flanks outside the central ±20-ms window, yielding a *z*-scored estimate of peak prominence. A positive peak reflects an excitatory coupling, whereas a negative peak indicates an inhibitory interaction. The time lag of the peak reveals the directionality of the connection, thereby identifying the source and target neuron. Across all positive and negative peaks, we separately determined the 99th percentile of their peak prominences, which served as significance thresholds (threshold for positive peaks, 7.04; for negative peaks, −5.0). We only further considered CCGs surpassing one of these thresholds. To avoid correlations as a result of spike-sorting artifacts, we excluded neuron pairs separated by <50 μm. To determine the directionality of coupling, we also excluded pairs with a time lag of 0 ms. Additionally, we measured the half-width of the negative peaks and excluded CCGs with a half-width of <1.5 ms. As the absolute significance threshold for negative peaks was lower than for positive peaks, such narrow peaks are more likely to reflect random coincidences rather than true interactions. Next, we only considered CCGs for which the peak lay within −6 ms to 6 ms. This threshold was chosen because the distribution of the absolute lag times sharply dropped beyond 6 ms (Extended Data Fig. 4d). Second, imposing a delay of 6 ms, we aimed to minimize potential polysynaptic connections^44^.

A concern for positive peaks in the CCGs is that they may arise from common input rather than reflecting directed synaptic interactions^73^ (Extended Data Fig. 4c and Supplementary Fig. 4). Because of synaptic transmission delays, excitatory synaptic interactions are expected to produce CCG peaks at nonzero time lags and low correlation for a time lag of 0 ms. To reduce common-input-driven CCGs in the dataset, we excluded positive CCGs for which the CCG value at 0 ms (CCG_0_) exceeded one-fourth of the peak CCG value (CCG_0_ / CCG_peak_ > 1/4). With this criterion, of 67 source neurons with more than one target neuron, 3 source neurons (4.48%) showed CCGs with opposing signs. As the percentage of coupled pairs using this approach is typically low (for example, <1% in cortex^44^), we included for the functional connectivity analysis the mice from all cohorts, including Neuropixels recordings in wild-type mice, dmM GAD2, vM VGLUT2, mPFC→LH stimulation experiments and eYFP control experiments (Supplementary Table 2).

### Analysis of coupling frequency

To determine whether specific pairs of neuron types occurred more frequently than expected by chance, we performed a shuffle-based permutation analysis. For both inhibitory and excitatory pairs, we first counted the observed number of neuron pairs belonging to each of the four possible categories (c2− ~ c2+, c2+ ~ c2−, c2− ~ c2−, c2+ ~ c2+). To generate a null distribution for each pair type, we performed 1,000 shuffles. In each shuffle, neuron pairs were sampled while preserving mouse identity: a mouse was randomly selected, and two distinct neurons were drawn without replacement from that mouse. This process was repeated until the number of sampled pairs matched the total number of observed pairs (across all four categories). For each shuffle, we then counted the frequency of each pair-type category. Two-tailed *P* values were computed by comparing the observed count for each pair type to its null distribution; pair types were considered significantly overrepresented or underrepresented if their observed count fell outside the 95% CI of the null distribution (that is, below the 2.5th or above the 97.5th percentile). *P* values were Bonferroni-corrected for multiple comparisons.

### Regional pairwise connection probability analysis

To estimate pairwise connection probabilities between brain regions, we first grouped anatomical regions into three major classes: pons (PRN, mP and dlP), raphe nuclei (DR, CS and RPO) and midbrain (PAG, MRN and vmMB). Source neurons were further subdivided into four categories based on their coupling sign and PC2 tuning: excitatory c2+, excitatory c2−, inhibitory c2+ and inhibitory c2− neurons. For each region pair and source-neuron category, we counted the number of functional connections between simultaneously recorded neuron pairs. Connection probabilities were computed by dividing the number of observed connections (for a given region pair and source-neuron category) by the total number of simultaneously recorded neuron pairs falling into the corresponding region pair and with the same PC2 source-neuron tuning (c2+ or c2−). As for functionally connected pairs, we only counted simultaneously recorded neuron pairs separated by more than 50 μm. To assess whether connection probabilities were enriched for specific region pairs, we compared the observed counts against a global baseline connection probability for each source-neuron category. The baseline probability for a given source-neuron category was defined as the total number of functional connections across all regions divided by the total number of simultaneously recorded neuron pairs with the same PC2 source-neuron tuning (Extended Data Fig. 4i). Statistically significant enrichment in connection probability was assessed using exact one-sided binomial tests comparing the observed counts to the baseline probability. *P* values were corrected for multiple comparisons using the Benjamini–Hochberg false discovery rate procedure, and CIs were determined using the Wilson score method (Supplementary Table 1).

### Statistics and reproducibility

No statistical methods were used to predetermine sample sizes, but our sample sizes are similar to those reported in previous publications using comparable methods^74^. Animals were not randomly assigned to experimental groups, and investigators were not blinded to group allocation during experiments or outcome assessment. We histologically verified that the location of Neuropixels probes and optic fiber implants were consistent across all animals in an experimental group. Representative histology images thus reflect the findings for all animals belonging to the same group. Recordings were excluded if the animal failed to sleep during the recording, if the number of recorded units was extremely low or if the Neuropixels probe was misplaced. In optogenetic experiments, mice were excluded if no viral expression was detected.

Statistical analyses were performed using the Python module pingouin (https://pingouin-stats.org/). All statistical tests were two-sided and a *P* value < 0.05 was considered as significant, unless stated otherwise. Data were compared using *t*-tests or ANOVA followed by pairwise tests with multiple-comparison correction. Normality was assessed with the Shapiro–Wilk test. When data were not normally distributed, non-parametric tests (Wilcoxon signed-rank or Mann–Whitney *U*-test) were used. For normally distributed data, pairwise comparisons were performed with *t*-tests, with Welch’s correction applied to unpaired tests in cases of unequal variances or sample sizes. For ANOVA, we used the Levene test for equal variances. For mixed and RM ANOVA, Mauchly’s test was applied to check the sphericity of the data. In case sphericity was violated, *P* values were corrected using Greenhouse–Geisser correction. KDE plots were fitted and plotted using Seaborn’s kdeplot function (https://seaborn.pydata.org/). Box plots were used to illustrate the distribution of data points. The upper and lower edges of the box correspond to the quartiles (25th and 75th percentile) of the dataset and the horizontal line in the box depicts the median, while the whiskers indicate the remaining distribution, except for outliers, that is, points smaller than the 25th percentile minus 1.5 times the interquartile range or larger than the 75th percentile plus 1.5 times the interquartile range. Outliers are depicted as open circles. Statistical test results are included in the corresponding figure legends and are detailed in Supplementary Table 1.

### Reporting summary

Further information on research design is available in the Nature Portfolio Reporting Summary linked to this article.

## Online content

Any methods, additional references, Nature Portfolio reporting summaries, source data, extended data, supplementary information, acknowledgements, peer review information; details of author contributions and competing interests; and statements of data and code availability are available at 10.1038/s41593-026-02314-z.

## Supplementary information

Supplementary Information Supplementary Figs. 1–4, Supplementary Tables 2 and 3 and Legend for Supplementary Video 1.

Reporting Summary

Supplementary Table 1 Statistical results for figures and Extended Data figures.

Supplementary Video 1 Trajectory of population activity in the state space.

## Source data

Source Data Fig. 1 Source data for Fig. 1.

Source Data Fig. 2 Source data for Fig. 2.

Source Data Fig. 3 Source data for Fig. 3.

Source Data Fig. 4 Source data for Fig. 4.

Source Data Fig. 5 Source data for Fig. 5.

Source Data Fig. 6 Source data for Fig. 6.

Source Data Extended Data Fig. 1 Source data for Extended Data Fig. 1.

Source Data Extended Data Fig. 3 Source data for Extended Data Fig. 3.

Source Data Extended Data Fig. 4 Source data for Extended Data Fig. 4.

Source Data Extended Data Fig. 5 Source data for Extended Data Fig. 5. The full raw data for Extended Data Fig. 5c can be found at https://doi.org/10.5281/zenodo.19462601.

Source Data Extended Data Fig. 6 Source data for Extended Data Fig. 6.

Source Data Extended Data Fig. 7 Source data for Extended Data Fig. 7.

Source Data Extended Data Fig. 8 Source data for Extended Data Fig. 8.

Source Data Extended Data Fig. 9 Source data for Extended Data Fig. 9.

Source Data Extended Data Fig. 10 Source data for Extended Data Fig. 10.

## Data Availability

Neuropixels datasets generated as part of this study are available on Zenodo via 10.5281/zenodo.19462601 (ref. ^75^). Source data are provided with this paper.

## Code availability

Code for sleep annotation, analysis of sleep data and EEG/EMG recordings is available on GitHub via https://github.com/tortugar/Lab/. Code for analysis of Neuropixels data is available at https://github.com/tortugar/Npx/.
