# Supplementary material for: Low-dimensional population dynamics in the brainstem gate REM sleep
Source: Nat Neurosci. 2026 May 25;29(7):1625–37. doi: 10.1038/s41593-026-02314-z (PMC13270127; doi:10.1038/s41593-026-02314-z)
Supplement: Supplementary file 1 — Supplementary Figs. 1–4, Supplementary Tables 2 and 3 and Legend for Supplementary Video 1. [file 41593_2026_2314_MOESM1_ESM.pdf]

nature neuroscience

Supplementary information

https://doi.org/10.1038/s41593-026-02314-z

Low-dimensional population dynamics in the brainstem gate REM sleep

In the format provided by the authors and unedited

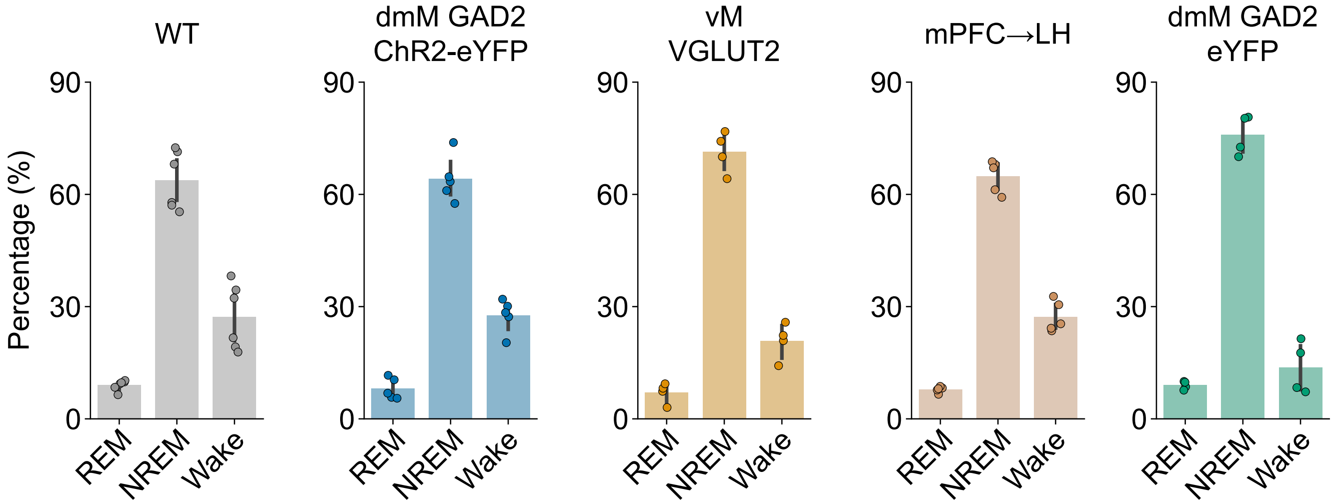

Supplementary Figure 1. Sleep-wake states in different experimental cohorts.

Percentages of sleep-wake states during Neuropixels recordings for the different experimental cohorts. Wild-type (WT), n = 6; dorsomedial medulla (dmM) GAD2 ChR2-eYFP, n = 5; ventral medulla (vM) VGLUT2, n = 4; mPFC→LH, n = 5; dmM GAD2 eYFP, n = 4 mice. Error bars, 95% confidence intervals (CIs).

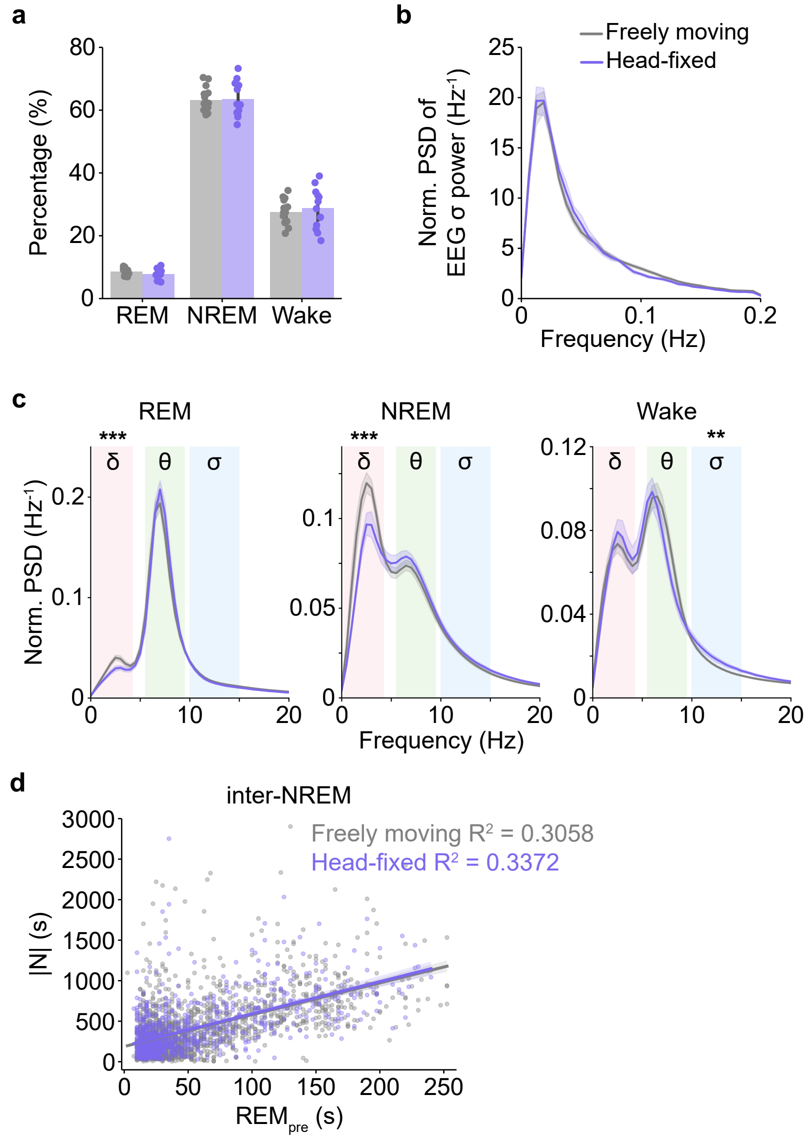

Supplementary Figure 2. Sleep in freely-moving and head-fixed mice.

(a) Percentages of sleep-wake states in head-fixed mice and freely-moving mice. Mixed ANOVA with brain state as within factor and recording condition (head-fixed vs freely-moving) as between factor; main effect of condition, F(1, 24) = 3.1804, P = 0.0872; interaction, F(2, 48) = 0.2396, P = 0.7879. Head-fixed mice, n = 12; freely moving mice, n = 14. Error bars, 95% CIs.

(b) PSD of normalized EEG σ (10 - 15 Hz) power during NREM sleep. Each PSD was normalized by dividing by its total power (area under the curve). Two-sided t-tests; maximum power, T(23.7474) = -0.6747, P = 0.5064; frequency with maximum power, T(23.8966) = 0.6792, P = 0.5036. Head-fixed mice, n = 12; freely-moving mice, n = 14. Shadings, 95% CIs.

(c) Normalized power spectral densities (PSDs) of the EEG during Wake, NREM, and REM sleep in head-fixed and freely-moving mice. Each PSD was normalized by dividing by its total power (area under the curve). Mixed ANOVA with frequency bands (δ, θ, and σ) as within factor and recording condition as between factor; REM, main effect of condition, F(1, 24) = 0.2237, P = 0.6405; interaction, F(2, 48) = 11.0664, P = 0.0001; two-sided t-tests with Bonferroni correction;

δ, T(20.3061) = -4.4569, P = 0.0001; θ, T(18.8709) = 2.1344, P = 0.1384; σ, T(18.2674) = -2.4120, P = 0.0798; NREM, main effect of condition, F(1, 24) = 15.0193, P = 0.0007; interaction, F(2, 48) = 23.9934, P = 0.0000; two-sided t-tests with Bonferroni correction; δ, T(23.0187) = -5.5144, P = 0.0000; θ, T(23.9524) = 2.0433, P = 0.1565; σ, T(22.6821) = 1.9967, P = 0.1740; Wake, main effect of condition, F(1, 24) = 0.1524, P = 0.6997; interaction, F(2, 48) = 5.1680, P = 0.0098; two-sided t-tests with Bonferroni correction; δ, T(18.9491) = 0.2300, P = 1.0000; θ, T(19.8064) = -2.4843, P = 0.0662; σ, T(13.4731) = 4.1887, P = 0.0030. Frequency bands: δ, 0.5 - 4.5 Hz; θ, 6.0 - 9.5 Hz; σ, 10 - 15 Hz. Head-fixed mice, n = 12; freely-moving mice, n = 14. Shadings, 95% CIs. ***P<0.001.

(d) Scatter plot with preceding REM sleep duration $\mathrm{REM}_{\mathrm{pre}}$ on the x-axis and subsequent duration of NREM sleep ($|\mathbf{N}|$) in the following inter-REM interval on the y-axis. Linear regression fits are shown as solid lines for each group. Linear regression; head-fixed: r^2 = 0.34, P $<$ 0.0001; freely-moving: r^2 = 0.31, P $<$ 0.0001; linear mixed-effects regression; intercept, P = 0.91; slope, P = 0.97; head-fixed mice, n = 891 inter-REM intervals from 12 mice; freely-moving mice, n = 1540 inter-REM intervals from 14 mice. Shadings, 95% CIs.

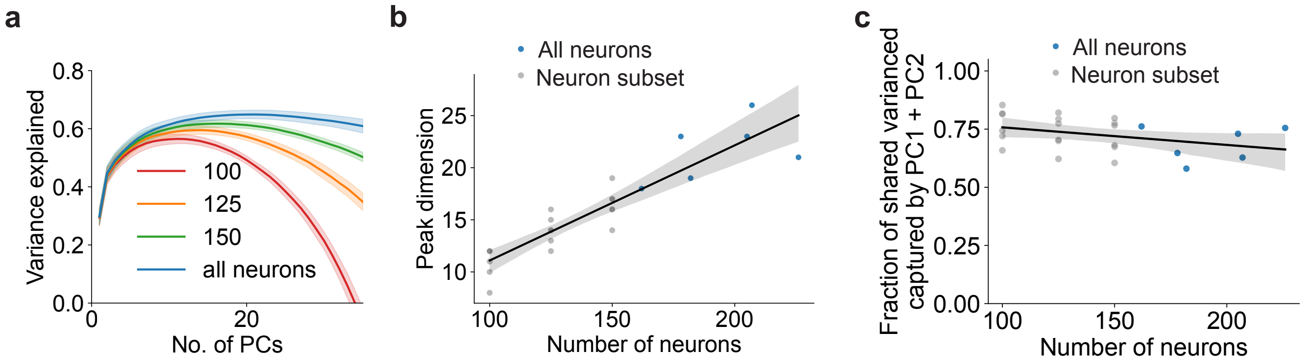

Supplementary Figure 3. Dependence of population dimensionality and shared variance explained by P1 and PC2 on neuron number.

(a) Relationship between the number of PCs and cross-validated variance explained for increasing numbers of simultaneously recorded neurons. We randomly selected fixed-size subsets of neurons (100, 125, or 150 neurons) from each recording and then performed the cross-validated variance explained analysis as in **Extended Data Fig. 1d**. Shadings, s.e.m.; n = 6 WT mice.

(b) Relationship between the number of simultaneously recorded neurons and the peak dimensions (number of PCs for which the variance explained curve in **a** reached its maximum. The dimensionality of the population activity increased with increasing number of neurons. Subsampled recordings are shown in gray; recordings with all neurons are shown in blue. Linear regression, P = 4.18e-10, r^2 = 0.8361, slope = 0.1105, 95%CI = [0.089, 0.13]. Shadings, 95% CI.

(c) Fraction of the shared variance explained by PC1 and PC2. Despite an increase in the estimated dimensionality with increasing neuron numbers, the fraction of shared variance captured by the first two PCs remained nearly constant across population sizes. Linear regression, P = 0.078, r^2 = 0.13, 95% CI = [-0.0016, 9.33e-05]. Shadings, 95%CI.

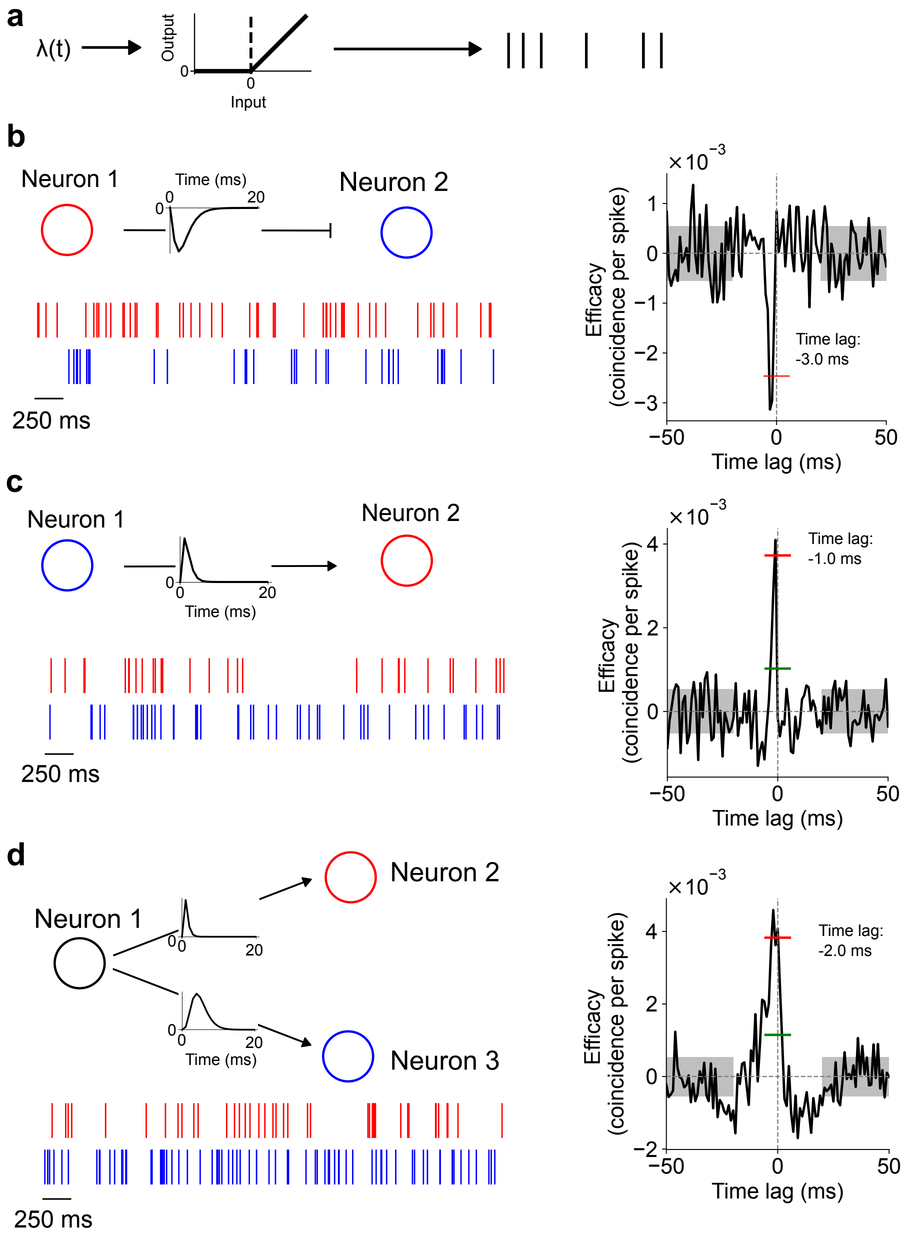

Supplementary Figure 4. Simulation of functional connections between pairs of neurons.

(a) Schematic of Poisson neuron. At each time point the probability of firing a spike depends on the instantaneous firing rate $\lambda(t)$. The input to the neuron is passed through a linear rectifier $r(\cdot)$. The resulting output is transformed into a spike train (1 ms binning) following the Poisson distribution with rate $r(\lambda(t)) \cdot dt$ with $dt = 1\,ms$.

(b) *Top left*, schematic illustrating inhibition of neuron 2 by neuron 1. Both neurons have a baseline firing rate of 10 Hz. The inhibitory interaction between the two neurons is modeled using a negative linear coupling filter. Each time neuron 1 fires a spike, the coupling filter is added to the future 20 ms of neuron 2's firing rate. Mathematically, the spike train of neuron 1 is convolved with the coupling filter and added to the rate of neuron 2. *Bottom left*, example of simulated spike trains. *Right*, jitter-corrected cross-correlogram (CCG). Spike trains were discretized in 1-ms bins and each lasted for 2 hours. The red line indicates the negative threshold that a peak in the

CCG had to cross to be considered significant (same as for experimental data **in Fig. 3**). The gray shadings indicate the baseline noise (1 std) in the CCG flanks.

(c) Excitatory interaction between two Poisson neurons modeled using a positive coupling filter. *Left,* schematic of the model and simulated spike trains. *Right,* CCG of the two neurons. To be considered as a direct excitatory interaction from neuron 1 to 2, the CCG peak had to occur at a non-zero time lag crossing the red line and the CCG value at time lag 0 ms had to be smaller than the green line, i.e. $CCG_{t=0}/CCG_{peak} < 1/4$. For the shown example, $CCG_0/CCG_{peak} = -0.037$.

(d) Common input to two neurons. Although there is no direct interaction between neurons 2 and 3, their spike trains are positively correlated due to common input from neuron 1. If the inputs, modeled using the coupling filters, from neuron 1 to the two postsynaptic neurons are not perfectly matched, the peak in the CCG can be asymmetric with its maximum at a non-zero lag time. Thus, a non-zero lag alone does not rule out common input as the cause of a positive correlation between two neurons. Given synaptic delays of several milliseconds, we included the CCG value at time lag 0 ms as an additional criterion: if a presynaptic neuron directly excites a postsynaptic neuron with some delay, the CCG should peak at a non-zero time lag, *and* its value at t = 0 ms should be close to 0. According to this criterion, the CCG between neurons 2 and 3 would not be classified as a direct excitatory connection, because $CCG_0/CCG_{peak} = 0.89$.

cohort	mouse	gender	PAG	MRN	vmMB	DR	CS	RPO	dlP	mP	PRN
WT	DL108	m	70	31	0	0	0	0	22	0	39
WT	DL158	m	80	0	0	0	0	0	102	0	0
WT	DL159	m	115	0	63	12	0	7	0	10	0
WT	DL161	m	50	41	0	0	0	0	19	0	116
WT	DL172	m	15	0	29	76	20	31	0	34	0
WT	DL194	m	51	0	22	46	12	31	0	16	0
GAD2	DL177	m	12	19	34	20	23	0	0	21	0
GAD2	DL207	m	11	31	0	0	0	0	5	0	66
GAD2	DL231	m	72	18	5	0	0	0	0	0	68
GAD2	DL240	m	35	41	0	0	0	0	6	0	71
GAD2	DL249	m	1	0	12	12	15	27	0	28	0
eYFP	JK459	m	19	0	29	0	38	0	0	0	0
eYFP	JK460	m	30	20	8	0	0	0	36	0	2
eYFP	JK461	m	46	69	0	0	0	0	7	0	68
eYFP	JK462	f	16	0	20	68	42	0	0	0	0
Vglut2	JK482	f	55	8	6	17	58	0	0	0	0
Vglut2	JK487	m	16	1	0	0	0	0	33	0	2
Vglut2	JK493	m	8	18	4	0	47	0	0	12	0
Vglut2	JK493b	m	5	0	12	11	0	0	0	60	0
Vglut2	JK496	m	15	8	6	0	1	0	0	10	34
PFC	J978	m	39	40	0	0	0	0	2	0	146
PFC	J978b	m	73	52	0	0	0	0	16	0	73
PFC	J980	m	58	0	23	0	11	40	49	0	0
PFC	J980b	m	2	0	4	12	23	41	65	0	47
PFC	J986	m	1	26	3	0	29	0	66	0	25
PFC	J987	m	0	30	5	0	47	0	41	0	59
PFC	J998	m	23	16	10	12	91	0	33	0	0
PFC	J998b	m	2	0	30	8	64	0	102	0	0

Supplementary Table 2. Counts of units per brain region and mouse.
In a subset of mice we performed two recordings; the second recording in a mouse is indicated by the suffix ’b’ in the mouse name.

	Exc c2+	Exc c2-	Inh c2+	Inh c2-	Total
MRN	3	3	4	9	19
PAG	5	15	3	9	32
vmMB	2	2	0	13	17
DR	4	2	0	0	6
RPO	2	4	1	5	12
CS	5	4	5	22	36
mP	5	7	5	7	24
dlP	0	3	0	3	6
PRN	11	23	12	25	71
Total	37	63	30	93	223

Supplementary Table 3. Counts of excitatory and inhibitory source neurons across brain regions.

Counts of putative excitatory and inhibitory source neurons with positive (c2+) or negative (c2−) PC2 tuning are shown for each recorded brain region; n = 100 excitatory and n = 123 inhibitory source neurons in 24 mice.

Supplementary Video 1. Visualization of population trajectory during NREM→REM→Wake transition.

The population activity across all neurons (rows) is represented as heatmap (left). The color-code of the trajectory in the state space (right) encodes time.
