# Supplementary material for: Low-dimensional population dynamics in the brainstem gate REM sleep
Source: Nat Neurosci. 2026 May 25;29(7):1625–37. doi: 10.1038/s41593-026-02314-z (PMC13270127; doi:10.1038/s41593-026-02314-z)
Supplement: Supplementary file 2 — Reporting Summary [file 41593_2026_2314_MOESM2_ESM.pdf]

nature portfolio

Corresponding author(s): Weber

Last updated by author(s): Jan 16, 2026

Reporting Summary

Nature Portfolio wishes to improve the reproducibility of the work that we publish. This form provides structure for consistency and transparency in reporting. For further information on Nature Portfolio policies, see our Editorial Policies and the Editorial Policy Checklist.

Statistics

For all statistical analyses, confirm that the following items are present in the figure legend, table legend, main text, or Methods section.

n/a	Confirmed	
☐	☒	The exact sample size (*n*) for each experimental group/condition, given as a discrete number and unit of measurement
☐	☒	A statement on whether measurements were taken from distinct samples or whether the same sample was measured repeatedly
☐	☒	The statistical test(s) used AND whether they are one- or two-sided<br>*Only common tests should be described solely by name; describe more complex techniques in the Methods section.*
☒	☐	A description of all covariates tested
☐	☒	A description of any assumptions or corrections, such as tests of normality and adjustment for multiple comparisons
☐	☒	A full description of the statistical parameters including central tendency (e.g. means) or other basic estimates (e.g. regression coefficient) AND variation (e.g. standard deviation) or associated estimates of uncertainty (e.g. confidence intervals)
☐	☒	For null hypothesis testing, the test statistic (e.g. *F*, *t*, *r*) with confidence intervals, effect sizes, degrees of freedom and *P* value noted<br>*Give P values as exact values whenever suitable.*
☒	☐	For Bayesian analysis, information on the choice of priors and Markov chain Monte Carlo settings
☒	☐	For hierarchical and complex designs, identification of the appropriate level for tests and full reporting of outcomes
☐	☒	Estimates of effect sizes (e.g. Cohen's *d*, Pearson's *r*), indicating how they were calculated

Our web collection on statistics for biologists contains articles on many of the points above.

Software and code

Policy information about availability of computer code

Data collection	EEG/EMG and Neuropixels recordings were performed using OpenEphys (version 0.4.6). Spikes were sorted using KiloSort2 (https://github.com/MouseLand/Kilosort/tree/kilosort2) and Kilosort4 (https://github.com/MouseLand/Kilosort). Clusters identified by Kilosort were manually curated in Phy (https://github.com/cortex-lab/phy). Neuropixels probe tracts were reconstructed using the software SHARP-track (https://github.com/cortex-lab/allenCCF). Neuropixels channels were aligned to anatomical features along the probe trajectory using the International Brain Lab (IBL) electrophysiology alignment tool (https://github.com/int-brain-lab/iblapps/tree/master/atlaselectrophysiology).
Data analysis	All sleep data were analyzed using custom python code (python 3.9, https://github.com/tortugar/Lab/blob/master/PySleep). Neuropixels data were analyzed using custom python code (https://github.com/tortugar/Npx). A demo code and dataset is available at https://github.com/tortugar/Npx/blob/main/basic_analysis_howto.ipynb. Visualization of probe trajectories and location of recorded neurons in the 3D Allen Mouse Brain Reference Atlas was performed using Urchin (https://github.com/VirtualBrainLab/Urchin).

For manuscripts utilizing custom algorithms or software that are central to the research but not yet described in published literature, software must be made available to editors and reviewers. We strongly encourage code deposition in a community repository (e.g. GitHub). See the Nature Portfolio guidelines for submitting code & software for further information.

Data

Policy information about availability of data

All manuscripts must include a data availability statement. This statement should provide the following information, where applicable:

- Accession codes, unique identifiers, or web links for publicly available datasets
- A description of any restrictions on data availability
- For clinical datasets or third party data, please ensure that the statement adheres to our policy

Neuropixels data sets generated as part of this study are available at Zenodo: https://doi.org/10.5281/zenodo.19462601 (ref. 75).

Research involving human participants, their data, or biological material

Policy information about studies with human participants or human data. See also policy information about sex, gender (identity/presentation), and sexual orientation and race, ethnicity and racism.

Reporting on sex and gender	n/a
Reporting on race, ethnicity, or other socially relevant groupings	n/a
Population characteristics	n/a
Recruitment	n/a
Ethics oversight	n/a

Note that full information on the approval of the study protocol must also be provided in the manuscript.

Field-specific reporting

Please select the one below that is the best fit for your research. If you are not sure, read the appropriate sections before making your selection.

☒ Life sciences ☐ Behavioural & social sciences ☐ Ecological, evolutionary & environmental sciences

For a reference copy of the document with all sections, see nature.com/documents/nr-reporting-summary-flat.pdf

Life sciences study design

All studies must disclose on these points even when the disclosure is negative.

Sample size	No statistical methods were used to predetermine sample sizes but our sample sizes were similar to those reported in previous publications using comparable methods (Oesch et al., 2023, PNAS, PMID: 32732431).
Data exclusions	Recordings were excluded if the animal failed to sleep during the recording, if the number of recorded units was extremely low, or if the Neuropixels probe was misplaced. In optogenetic experiments, mice were excluded if no viral expression was detected.
Replication	Neuropixels recordings were performed one at a time. All key findings (shape of PCs and state space, subclasses of neurons, and effects of optogenetic experiments) were replicated across individual animals.
Randomization	Animals were not randomly assigned to experimental groups. For optogenetic open-loop stimulation (60 s protocol), the timing of laser stimulation was randomized.
Blinding	The investigators were not blind to the group allocation during the experiments and outcome assessment. For sleep scoring, annotators were blinded to the timing of the laser, and further analysis of the single unit data and EEG/EMG signals was conducted by computer programs in an automated and unbiased way.

Reporting for specific materials, systems and methods

We require information from authors about some types of materials, experimental systems and methods used in many studies. Here, indicate whether each material, system or method listed is relevant to your study. If you are not sure if a list item applies to your research, read the appropriate section before selecting a response.

Materials & experimental systems

n/a	Involved in the study	
	☒	Antibodies
☒	☐	Eukaryotic cell lines
☒	☐	Palaeontology and archaeology
	☒	Animals and other organisms
☒	☐	Clinical data
☒	☐	Dual use research of concern
☒	☐	Plants

Methods

n/a	Involved in the study	
☒	☐	ChIP-seq
☒	☐	Flow cytometry
☒	☐	MRI-based neuroimaging

Antibodies

Antibodies used

anti-GFP chicken antibody (1:1000, Aves Lab, GFP-1020)
anti-chicken-AlexaFluor488 (1:500, Jackson ImmunoResearch Laboratories, 703-545-155)
anti-chicken-AlexaFluor594 (1:500, Jackson ImmunoResearch Laboratories, 703-585-155)

Validation

All antibodies have been used and validated in previous studies (e.g., Yuan et al., 2016, PMID: 27822503).

Animals and other research organisms

Policy information about studies involving animals; ARRIVE guidelines recommended for reporting animal research, and Sex and Gender in Research

Laboratory animals

All experiments were performed in C57BL/6J mice (Jackson Laboratory; stock no. 000664), GAD2-IRES-Cre mice (stock no. 010802), and VGLUT2-IRES-Cre mice (Jackson Laboratory stock no. 016963) aged 8 – 12 weeks old at the point of surgery.

Wild animals

This study did not involve wild animals.

Reporting on sex

The sex of each animal is reported in Supplementary Table 2, with the majority being male. In our previous studies (Stucynski et al., 2022; PMID: 34735794; Hong et al., PMID: 37735498), we compared males and females and did not detect any sex-dependent differences in the effects of optogenetic stimulation of dmM GAD2 neurons or LH-projecting mPFC neurons on brain state.

Field-collected samples

This study did not involve samples collected from the field.

Ethics oversight

All animal care and experimental procedures were approved by the Institutional Animal Care and Use Committee (IACUC) at the University of Pennsylvania and conducted in accordance with the National Institutes of Health Office of Laboratory Animal Welfare Policy.

Note that full information on the approval of the study protocol must also be provided in the manuscript.

Plants

Seed stocks

n/a

Novel plant genotypes

n/a

Authentication

n/a
